# Therapeutic interventions alter ecological interactions among cystic fibrosis airway microbiota

**DOI:** 10.3389/fmicb.2023.1178131

**Published:** 2023-05-30

**Authors:** Pok-Man Ho, Rahan Rudland Nazeer, Martin Welch

**Affiliations:** Department of Biochemistry, University of Cambridge, Cambridge, United Kingdom

**Keywords:** cystic fibrosis, microbial ecology, Lotka-Volterra, gut-lung axis, pancrelipase, DNase

## Abstract

The airways of people with cystic fibrosis (*CF*) often harbor a diverse microbiota and in recent years, much effort has been invested in cataloguing these. In spite of providing a wealth of insight, this cataloguing tells us little about how the organisms interact with one another in the *CF* airways. However, such relationships can be inferred using the theoretical framework of the Lotka-Volterra (LV) model. In the current work, we use a generalized Lotka-Volterra model to interrogate the nationwide data collected and curated by the UK *CF* Registry. This longitudinal dataset (covering the period 2008–2020) contains annual depositions that record the presence/absence of microbial taxa in each patient, their medication, and their *CF* genotype. Specifically, we wanted to identify trends in ecological relationships between the *CF* microbiota at a nationwide level, and whether these are potentially affected by medication. Our results show that some medications have a distinct influence on the microbial interactome, especially those that potentially influence the “gut-lung axis” or mucus viscosity. In particular, we found that patients treated with a combination of antimicrobial agents (targeting the airway microbiota), digestive enzymes (assisting in the assimilation of dietary fats and carbohydrates), and DNase (to reduce mucus viscosity) displayed a distinctly different airway interactome compared with patients treated separately with these medications.

## Introduction

Cystic fibrosis (*CF*) is a life-limiting disease which has been estimated to affect around 100,000 people worldwide (Guo et al., 2022), and around 11,000 people in the UK (Taylor-Robinson et al., 2018). The disease is caused by defective targeting or activity of the cystic fibrosis conductance regulator (CFTR); a chloride/bicarbonate channel located in the epithelial cells of the GI tract and airways, among many other tissues (Gregory et al., 1990). Mutations in the CFTR give rise to defective Cl^−^ transport across the epithelial cell membrane. As a consequence of this, the distribution of water across the epithelium becomes imbalanced, leading to decreased hydration of the surrounding mucus layer (Chmiel and Davis, 2003; Cohen-Cymberknoh et al., 2011; Cohen and Prince, 2012; Cohen-Cymberknoh et al., 2013). The resulting mucosal dysbiosis and consequent airway “eutrophication” results in colonization by a variety of microbes, including Gram-positive, Gram-negative, mycobacterial and fungal species (Cox et al., 2010). Although more than 2000 different CFTR mutations have been associated with *CF* (De Boeck, 2020), in the Caucasian population (which are the most intensively studied cohort) the most prevalent CFTR mutation is the ∆F508 deletion (Guo et al., 2022). This impairs proper folding (and hence, targeting) of the protein (Rodrigues et al., 2008).

The airways of people with *CF* (pwCF) are initially colonized by opportunists such as *Staphylococcus aureus* and *Haemophilus influenzae* (Frayman et al., 2017). However, as they approach their ‘teens, pwCF acquire additional species, including *Pseudomonas aeruginosa*, *Stenotrophomonas maltophilia* (Cox et al., 2010), *Candida albicans* (Martínez-Rodríguez et al., 2022) and others. These respiratory infections lead to many *CF*-associated mortalities (Castellani and Assael, 2017), and a major goal of therapeutic interventions is to eradicate or manage these infections. Consequently, many pwCF are routinely treated with antibiotics. These antibiotics may be used on a day-to-day basis for chronic suppressive therapy (e.g., inhaled tobramycin) or may be deployed more intensively (e.g., intravenously) to help resolve the intermittent pulmonary exacerbations that often punctuate progression of the disease (Castellani and Assael, 2017). Although many of these antimicrobial interventions are “targeted” at the principal pathogen(s) present, such as *P. aeruginosa*, they almost certainly also affect the other microbiota present, leading to a wider remodeling of the community dynamics (Carmody et al., 2013). In addition to treatment with antimicrobials, many pwCF also take other therapeutics such as anti-inflammatories (Roesch et al., 2018), inhaled DNase (Hurt and Bilton, 2014), and nutritional supplements such as pancrelipase and vitamins (Brownell et al., 2019). Moreover, correctors and potentiators (collectively known as CFTR modulators) have recently been introduced to [partially] “correct” the functional defects brought about by the CFTR mutation(s) (Gramegna et al., 2020). Correctors assist in CFTR folding (ensuring that at least some of the protein reaches the epithelial membrane in the correct configuration) whereas potentiators lock the correctly-targeted CFTR in an “open” (active) conformation. The composition (Cox et al., 2010; Carmody et al., 2013) and inter-species interactions (Lindsay and Hogan, 2014; Reece et al., 2021) among the microbiota that occupy the *CF* airways may therefore be influenced by multiple variables.

Approaches aimed at understanding the ecological interactions between microbes in the *CF* airways are currently very limited, and remain dominated by the study of pairwise interactions between species (Hogan and Kolter, 2002; Lindsay and Hogan, 2014; Reece et al., 2021). As a result, the survival strategies used by microorganisms in complex, polymicrobial communities remain largely uncharacterized. This notwithstanding, some progress has been made. One pioneering effort in this regard has been development of the so-called “climax-attack” model (CAM) (Conrad et al., 2013), introduced by Conrad et al. in 2013 to describe the *CF* microbiota succession dynamics following episodes of acute pulmonary exacerbation. The CAM is based around the generalized Lotka-Volterra (gLV) equations. The Lotka-Volterra (LV) model was introduced to describe the oscillating dynamics of predator–prey interactions in an ecosystem (Lotka, 1925; Volterra, 1926) (most famously, between lynx and hare populations in Canada around the turn of the 20th century). The basic LV model has since been modified to yield the gLV model (Hofbauer et al., 1987; Rand et al., 1994), which captures a wider variety of ecological interactions, including commensalism, mutualism, amensalism and competition, among others. However, although the CAM is a very useful tool for describing the succession dynamics following pulmonary exacerbation in individuals, it does not describe ecological trends in the wider population. In 2014, Whiteson et al. proposed an alternative ecological model to describe the dynamics of microbial colonization of the *CF* airways; the “Island Biogeography Model” (IBM) (Whiteson et al., 2014). Here, microbiota in the upper respiratory tract successively spread into the lower airway regions during colonization, giving rise to spatial heterogeneity. A competing model – “neutral theory” – assumes a more stochastic (homogenous) distribution of colonizing microorganisms (Huang et al., 2018). However, this proves to be a poor model of *CF* airway colonization. A common feature of the CAM, IBM and neutral models is that they all assume that pairs of organisms are characterized by just a single, fixed ecological relationship in a given environment.

We wondered whether ecological relationships between species in the *CF* airways may be fluid, given that the system is periodically challenged with antimicrobials and other interventions that likely give rise to population remodeling. To begin to address this question, we have developed a model, based on the gLV, to monitor changes in ecological relationships across discrete time windows. Our analysis is based on Bayesian Inference adaptive Markov Chain Monte Carlo (aMCMC) simulations using the gLV framework (as detailed in Supplementary Information Sections 2–5). We use this model to analyze the ecological interactions between microbial taxa recorded in the UK *CF* registry over the period 2008–2020. This dataset records the presence/absence of specified microbes (but not their individual titers or relative abundances), across a comprehensive swathe of the UK *CF* population. One limitation of this dataset is that “medications” are entered into the Registry throughout the year, and so represents a cumulative record of therapeutic intervention inclusive of, e.g., management antimicrobial therapies, CFTR modulators, and the aggressive antibiotic interventions used to treat the pulmonary exacerbation episodes which punctuate progression of the disease (Goss, 2019). For simplicity, we focus on those pwCF who are homozygous for the ∆F508 CFTR mutation, which is the most common allele encountered in the UK *CF* community. Using a rolling time window, our data show that the ecological relationships between taxa can (and do) change over time, and that even within a given timeframe, each taxonomic group can display signatures consistent with multiple ecological roles. Moreover, our data indicate that certain therapeutic interventions can have a significant impact on microbial interactions.

## Methods

**Figure 1:**
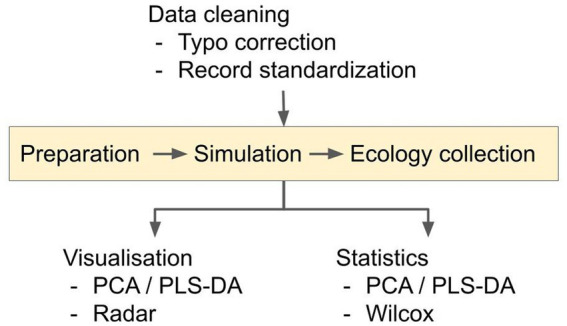
Overview of the analysis pipeline. The data cleaning converts microbial presence/absence data into a percentage-presence time-series for pwCF who are homozygous for the ∆F508 allele. Time-series were generated for each of the 10 microbial categories. Pairwise inter- and intra-taxa ecological interactions in the resulting time-series data were simulated using the Bayesian Inference and aMCMC gLV equations, as described in the Supplementary Information sections 2–5. Possible ecological interactions between- and within- microbial categories were then inferred for the respective medication groups.

We employed a three-step pipeline for data mining (Figure 1); data cleaning and preparation, data simulation and data collection. Raw data was obtained (with approval) from the UK *CF* Registry. The dataset comprised a presence/absence list of microbes in each patient, their CFTR genotype, and their recent history of medication. Although data can (and often is) entered into the Registry throughout the year, auto-populating, e.g., the “microbiology” and “medication” fields, some of the data is collected during detailed annual review encounters with each patient (annual reviews are implemented for all consenting adult *CF* patients). Consequently, the annual review combines a snapshot of each patient at the time of the annual review, combined with a portrait of their medication and microbiology over the preceding year. The annual review data are only collected when the patient is “well” i.e., during a period of stability, and not undergoing an exacerbation (although exacerbations are likely to have occurred during the previous year for many pwCF). Through active consultation, the Registry data have been designed to be standardized and comparable with longitudinal and cross-sectional databases internationally.

**Figure 2:**
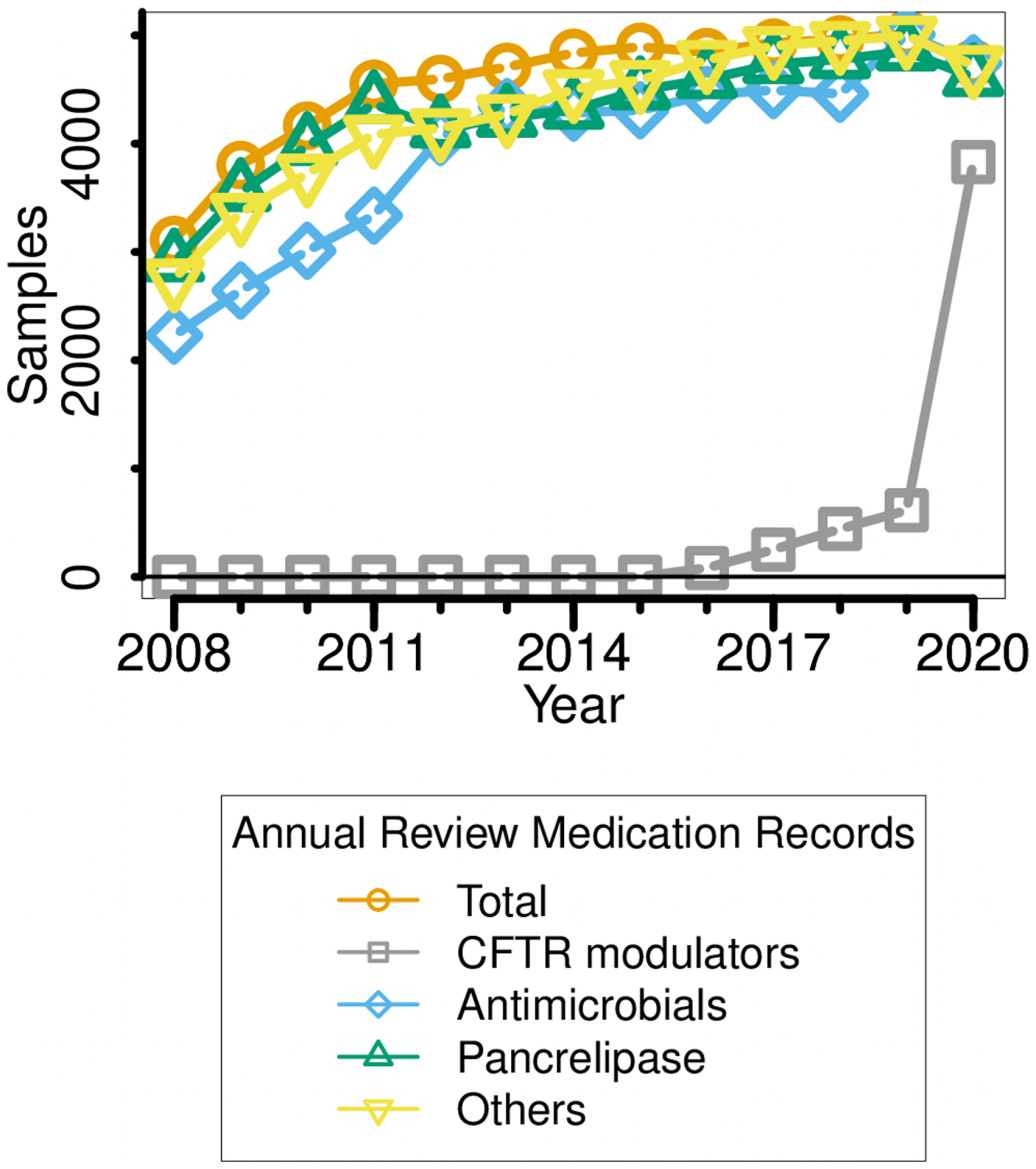
Medication types taken by pwCF ∆F508 over the period 2008–2020. Note that the data reported for a given year (e.g., 2014) refer to the medication(s) prescribed in the year leading up to that point (i.e., 2013–2014).

A preliminary inspection of the data indicated that the data would require “cleaning” before further analysis, to repair typos and other obvious transcription/data entry inaccuracies (e.g., spelling variations for species/taxa and treatment names). Typo correction comprised an automated Google search with manual verification, followed by cross-referencing with peer-reviewed taxonomy (Schoch et al., 2020), medication (e.g., Wishart et al., 2006) databases and the mutation list from the UK *CF* Registry. After cleaning, we further filtered the data to include only those individuals who are homozygous for the ∆F508 mutation. Microbial presence/absence data was extracted from the Registry. These data are mostly from culture-based analyses, and may be entered into the Registry at any time in the year leading up to the annual review. We note that a major weakness of the Registry data is that, due to the (largely historical) bias toward easy-to-culture species, they rarely record taxa that we now know to be abundant in many patients from, e.g., 16S rDNA analyses [e.g., the anaerobes (Thornton and Surette, 2021)]. Prior to 2013, the Registry records focused on where the sample was harvested (a *CF* center, “elsewhere,” or not recorded), but from 2013 onwards, these “location” data were replaced with “source of sample” (sputum, BAL, cough swab etc). After data cleaning, the presence of a given taxon was recorded as a “1” whereas the absence was recorded as a “0.” The microbial taxa (Supplementary Figure S1) were coagulated into 10 prevalent categories [based on them being present in a minimum of 5% of the sampled population in any given year (Supplementary Figure S2)]. The 10 microbial categories were *Aspergillus*, *Candida*, *Haemophilus*, mycobacteria, *Pseudomonas*, *Staphylococcus*, *Stenotrophomonas*, Yeast, “unidentified” and “others.” We next further categorized the data entries based on medication received. The database recorded 1,474 different medications/interventions; however, only a few of these were prescribed for most patients (Supplementary Figure S3). For simplicity, and to reduce computational complexity to a manageable level, we therefore focused on the most widely-used interventions (antimicrobial agents and pancrelipase), and on the category “CFTR modulators.” The latter was included in the analyses because although not all pwCF∆F508 received these drugs over the time period examined, changes in prescription policy now mean that almost all patients now do receive these drugs (Figure 2). Treatments not included in antimicrobials/pancrelipase/CFTR modulators were coagulated into “others,” which includes all other identified treatment classes, e.g., anti-depressant, anti-histamine, agonist etc. We then interrogated the dataset to establish which combinations of these four broad therapeutic intervention categories were used on a sufficiently large number of pwCF to allow robust simulations to be inferred. For inclusion in our analyses, we required each medication/combination to be used at least 5 times in a given year, and ≥ 30 times over all of the years examined (Supplementary Figure S4). Based on this, we included eight treatments/combinations in our analysis: “antimicrobial agents only,” “pancrelipase only,” “others only,” “antimicrobial agents + pancrelipase,” “antimicrobial agents + others,” “antimicrobial agents + pancrelipase + others,” “pancrelipase + others,” and “antimicrobial agents + pancrelipase + others + CFTR modulators.” The exact number of pwCF receiving each of these medications/combinations is indicated in the relevant graphs shown in Supplementary Figures S5–S12. [The category “antimicrobial agents + others + CFTR modulators” also satisfied the conditions for inclusion in the analyses. However, the data could not be modelled using the gLV]. After grouping the data, we carried out a time-series conversion from the coagulated individual-level data by calculating the proportion of pwCF in each category (considering microbial taxa and treatment) in the respective year. The resulting time-series data were then combined into a moving three-year window.

Following data cleaning, we calculated the interaction coefficients (between- and within- microbial categories) and also the colonization rates. Due to the paucity of experimental data relating to inter-species interactions at the [inter]national level, we set agnostic boundaries for the interaction coefficients. Colonization rates were initially optimized with rough estimates and then refined by mathematical optimization. This was done because statistical analysis of each colonization curve suggested a range of possible colonization rates. Once the boundaries for the colonization rate by each microbial category had been approximated, they were subsequently refined by mathematical optimization as described in the Supplementary Information. Hereafter, and for simplicity, unless otherwise indicated, we refer to each microbial category as “species.”

Once the parameters for the inter/intra-species/taxa interaction coefficient and colonization rate had been determined, we input these into the gLV model to generate simulations. Simulations were performed on the Cambridge High Performance Computing Cluster (CSD3 Skylake-himem cluster). For each of the 10 “microbial” time-series, 500,000 simulations were carried out on each of 7 replicates. These replicates each had the same input parameter values, but were allowed to follow different subsequent trajectories (i.e., a random forest decision tree). The different trajectories were initiated by designation of each replicate with a random seed number, which influences the subsequent aMCMC pathway within a Bayesian Inference framework. The top 0.1% of the resulting simulations [in terms of fit to the registry data (see Supplementary Figures S5–S12)] were used for downstream analysis. Specifically, the simulations were used to detect and test the different ecological interactions which best accounted for the observed dynamics in the data.

Ecological relationships for each pairwise species combination were deduced from the simulations that best fit the time-series data. Specifically, the interaction coefficients for species A and species B in the best-fit simulations each have a sign (+, 0 or -). These signs can be used to assign well-established ecological relationships: mutualism (+,+), commensal-host (+,0; commensalism), prey/host (+,-; predatory), commensal (0,+; commensalism), neutral/no interaction (0,0), harmed by (0,-; amensalism), predator/parasite (-,+; predatory), harming (-,0; amensalism) and competition (-,-). In essence, “+” indicates a net synergistic effect, “0” indicates a net neutral effect, and “-” indicates a net antagonistic effect.

Based on the inferred ecology, we carried out three types of statistical analysis, determining correlation, causation and multivariate clustering. In particular, we focused on understanding better how therapeutic interventions impact on the ecology of the *CF* airways. This could be inferred from the “robustness” of the gLV model outputs (which is reflected in the ability of the gLV to successfully model a given pairwise interaction). For example, for two species, if their ecological interaction does not change following a given treatment, the interaction is usually very successfully modelled by the gLV. However, if the treatment alters the interaction between these two species, this change in the ecology toward a more “mixed” set of interactions gives rise to a drop in the ability of the model to capture a given ecological interaction. Therefore, by examining whether there is any correlation (Spearman coefficient with Benjamini/Hochberg false discovery rate *p*-value correction) between the gLV simulation success rate and a given therapeutic intervention, we were able to quantitatively *correlate* changes in the ecology between different species through time. To test the *causality* of these changes, we applied linear regression. To detect the ecological distinctiveness between different medication groups, we applied multivariate clustering.

The simulations were associated with two independent variables (“therapeutic intervention” and “microbial category”) and one multi-dimensional dependent variable (the ecological interactions). To detect the influence of each independent variable, we used the Principal Components Analysis (PCA) and Partial Least Squares-Discriminant Analysis (PLS-DA, supervised by the independent variable of interest). To investigate the impact of therapy on ecological interactions, for each therapeutic intervention, we summarized the ecology of each species pair across all time windows. This yielded 440 outputs (45 inter- and 10 intra-taxon/species pairs for each of the 8 therapeutic interventions). To investigate the impact of taxonomy on ecological interactions, we likewise summarized the interactions from each taxonomic category across all time windows. This yielded 80 “taxonomic tags” (10 taxonomic categories for each of the 8 therapeutic interventions). To visualize the combined effect of the two independent variables, we employed a Hierarchical Cluster Analysis (HCA) on the 80 taxonomic tags.

## Results

### 1.1. Trends in medication

Between 2008 and 2020, there were several obvious changes in therapeutic intervention for pwCF who are homozygous for the ∆F508 allele. First, antimicrobial prescription rose sharply in the periods 2010–2012 and 2017–2018 (Figure 2). Indeed, by 2020, all sampled ∆F508 pwCF were receiving antimicrobial treatment. Second, in the period 2012–2018, a small but ever-increasing minority of ∆F508 pwCF received CFTR modulators (Kmietowicz, 2019; Smyth, 2020; Keogh et al., 2022). [Clinical trials with Ivacaftor/Kalydeco began in the UK in 2012] However, there was a sharp increase in CFTR modulator use in 2019, such that by the following year, >80% of pwCF ∆F508 were using these drug(s), concomitant with the introduction of NHS-funded Trikafta/Kaftrio in the UK *CF* community (Kmietowicz, 2019; Smyth, 2020).

### 1.2. Correlation of ecological predictability with medication

Ecological predictability is a good indicator of ecosystem stability; if inter-species interactions do not change over time, the system can be considered predictable and therefore, stable. To examine whether there was any correlation between ecological predictability (as assessed from the ability of the simulations to successfully capture pairwise interactions) and medication type, we calculated the success rate of the simulations in a rolling three-year window, and then rank ordered the outcomes to calculate the correlation coefficient (ρ). This revealed that none of the medication categories correlated with stability of the airway ecosystem (*data not shown*).

### 1.3. Causal effects of medication

Correlation does not necessarily imply causation. To establish possible causal associations between ecology and medication, we employed linear regression. Of the factors examined, CFTR modulators (*p* = 0.08) had a potentially distinct impact on ecological predictability (*data not shown*).

### 1.4. Impact of therapeutic intervention on ecological interactions

**Figure 3:**
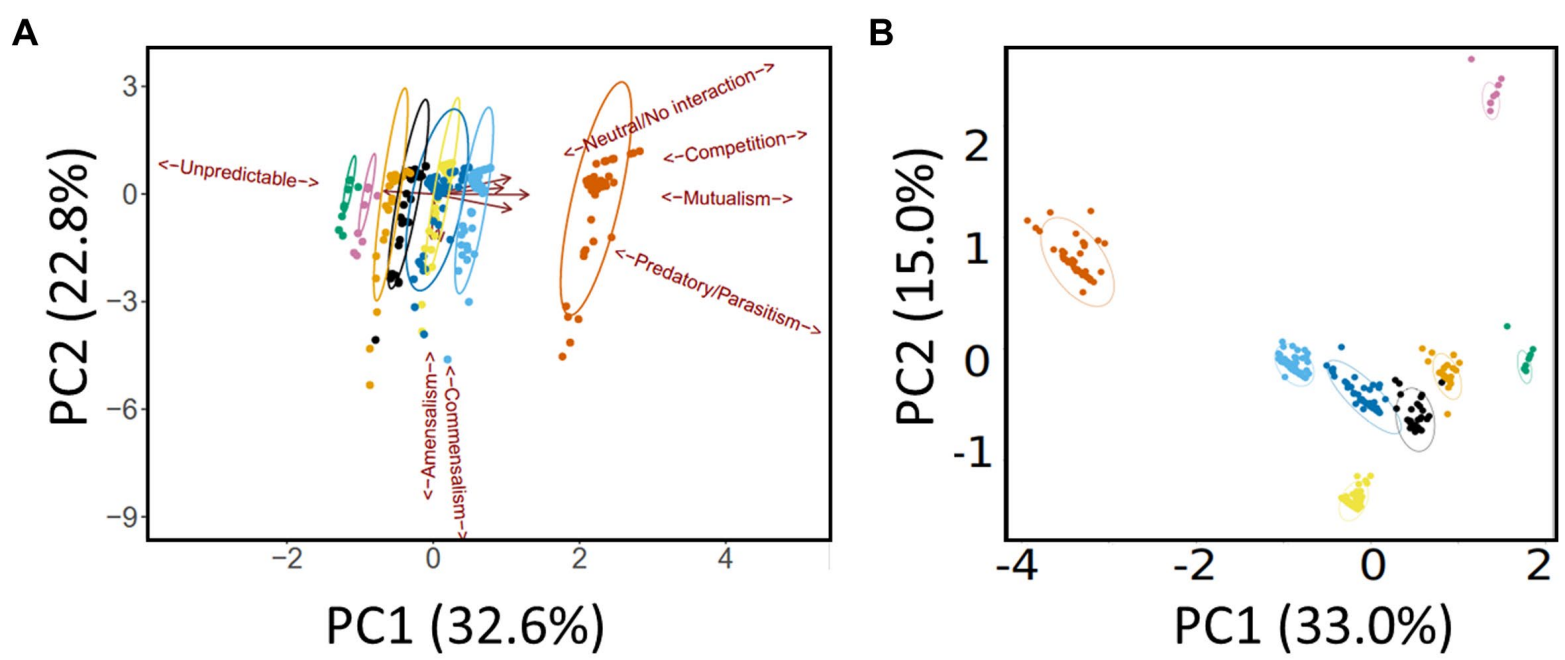
Multivariate analysis of the data. **(A)** Principal Components Analysis (PCA) scores plot. Trends associated with different types of ecological interaction (mutualism, commensalism etc) are indicated. **(B)** Partial Least Squares-Discriminant Analysis (PLS-DA) scores plot. Ellipses show 95% confidence boundaries. Key: 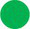
antimicrobials only, 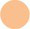 pancrelipase, 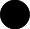 others, 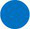 antimicrobials + pancrelipase, 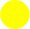 antimicrobials + others, 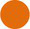 antimicrobials + pancrelipase + others, 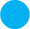 pancrelipase + others, 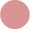 antimicrobial agents + pancrelipase + others + CFTR modulators.

As outlined in the Methods section, we defined 10 categories (“species”) of microbe. This yielded 10 × 10 (i.e., 100) possible inter- and intra-species interactions. Subtracting the 10 intra-species interactions and halving the resultant yielded 45 inter-species pairs. The gLV model was used to assess each of these pairwise interactions over the eight medication groupings (e.g., “antimicrobial agents only,” “antimicrobial agents + pancrelipase” etc) outlined in the Methods section. [We note that pwCF moved in and out of the different medication groupings based on clinical need, so membership of a given grouping is best thought of as “fluid” rather than having a fixed composition. However, the gLV model that we apply here is well-suited to an analysis of such a fluid cohort (Elton and Nicholson, 1942)]. This yielded a total of 440 possible intra- and inter-species pairwise interactions covering all treatment types [(45 + 10) × 8]. Given this large number of outputs, and to identify which medications had the greatest impact on pairwise interactions, we analyzed the data using principle components analysis (PCA). Specifically, the input for the PCA was the summary of ecological interactions (themselves, the summary of the best-fit simulations) for each pairwise species combination. The PCA scores plot is shown in Figure 3A (here, the different therapeutic interventions associated with the 440 possible pairwise interactions are designated with different colors). Perhaps the most obvious feature is that, independent of the therapeutic intervention, most of the model outputs strongly co-clustered, independent of medical intervention. However, there was some segregation of the clusters, and one treatment type (antimicrobial agents + pancrelipase + others) was clearly separated from the others along PC1 (corresponding to the mutualism/predation/parasitism/competition/neutral axis). To gain further insight into the impact of medications on the microbial interactome, we also analyzed the model outputs using a supervised approach, partial least squares-discriminant analysis (PLS-DA), with the supervised factor being type of medication (Figure 3B). This clearly segregated each therapeutic intervention, suggesting that, by-and-large, different treatments do indeed elicit distinct ecological behaviors in the microbiota. The clusters corresponding to treatments “antimicrobials + pancrelipase + others” in either the presence or the absence of CFTR modulators were particularly distinct from the others.

### 1.5. Impact of therapeutic intervention on the ecological interactions of different taxa

**Figure 4:**
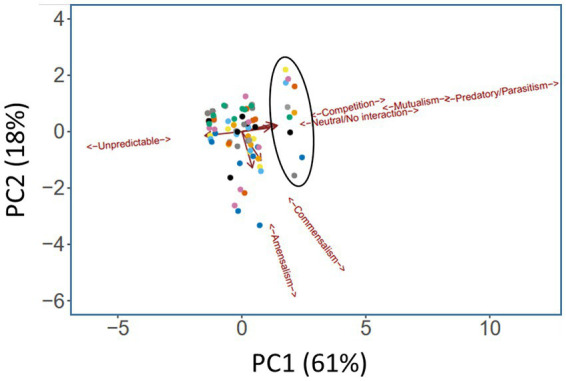
Principal Components Analysis (PCA) scores plot showing ecological role colored according to taxonomic category. Trends associated with different types of ecological interaction (mutualism, commensalism etc) are indicated. The ellipse highlights taxa in the medication category “antimicrobials + pancrelipase + others.” Key: 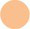 *Aspergillus* spp., 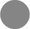 *Candida* spp., 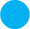 *Haemophilus* spp., 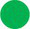 mycobacteria, 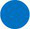 *Pseudomonas* spp., 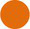 *Staphylococcus* spp., 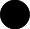 *Stenotrophomonas* spp., 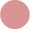 unidentified, 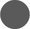 Yeast, 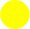 others.

The segregation in Figure 3 indicates that certain medications have a clear impact on the microbial interactome. To further investigate this, we next asked whether a similar conclusion could be drawn if the segregation was based on taxonomic category instead of therapeutic intervention. To do this, we summarized the cumulative pairwise interactome between each taxon and all other taxa (including itself) across all time windows. [An example of how this was done is shown in Supplementary Figure S13 for the *Pseudomonas*-Yeast interaction in the category treatment with “antimicrobial agents + pancrelipase + others.” These pairwise time-series data also highlighted that the taxa are rather fluid in terms of their ecological interactions, and that even a single pairwise interaction can manifest more than one ecological signature]. This summary approach yielded 80 “taxonomic tags” (10 taxa × 8 therapeutic intervention categories), and following PCA, these once again displayed a high degree of co-clustering with relatively little segregation based on taxonomic category (Figure 4). However, one group of tags were prominently displaced away from the main cluster, segregating along the mutualism/predation/parasitism/competition/neutral axis (PC1). Consistent with the outcome in Figure 3, the corresponding taxonomic tags (circled in Figure 4) were all from the category “antimicrobials + pancrelipase + others.” To visualize the data in an alternative way, we used hierarchical cluster analysis (HCA) to segregate the ecological interactions associated with each taxonomic tag (Supplementary Figure S14). The most obvious division segregated the taxa treated with “antimicrobials + pancrelipase + others” from the taxa treated with other therapeutic interventions.

**Figure 5:**
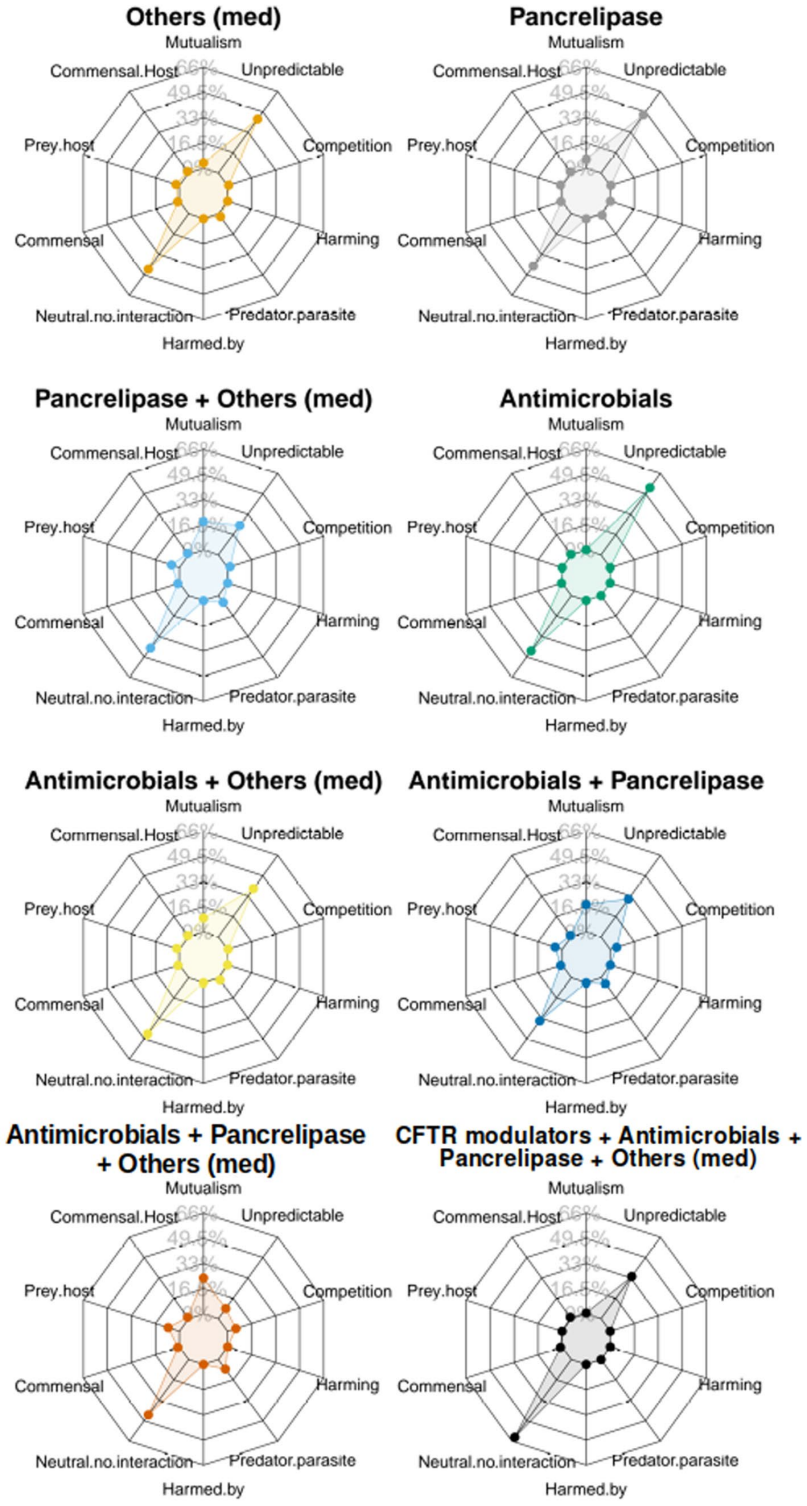
The figure shows a set of “radar plots” showing ecological interaction colored according to category of clinical intervention. Key: 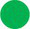 antimicrobials only, 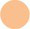 pancrelipase, 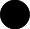 others, 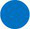 antimicrobials + pancrelipase, 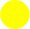 antimicrobials + others, 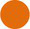 antimicrobials + pancrelipase + others, 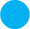 pancrelipase + others, 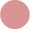 antimicrobial agents + pancrelipase + others + CFTR modulators.

To gain a more holistic view about the different medications, and in particular, how treatment with “antimicrobials + pancrelipase + others” affects taxonomic interactions, we sequentially summarized the data in Figure 4, using the approach shown in Supplementary Figure S13, to yield “radar plots.” Most of these display a “compass-like” signature (Figure 5), with the “compass needle” aligned along the neutral-unpredictable axis. However, treatment with “antimicrobials + pancrelipase + others” (and to a lesser extent, also with “antimicrobials + pancrelipase,” and “pancrelipase + others”) leads to a much more predictable ecology. The impact of treatment with “antimicrobials + pancrelipase + others” on the ecological interactions between individual taxa are shown in Supplementary Figure S15. We note that all of the taxa appear to be similarly affected, with a clear shift toward predation, mutualism and parasitism in all cases.

**Figure 6:**
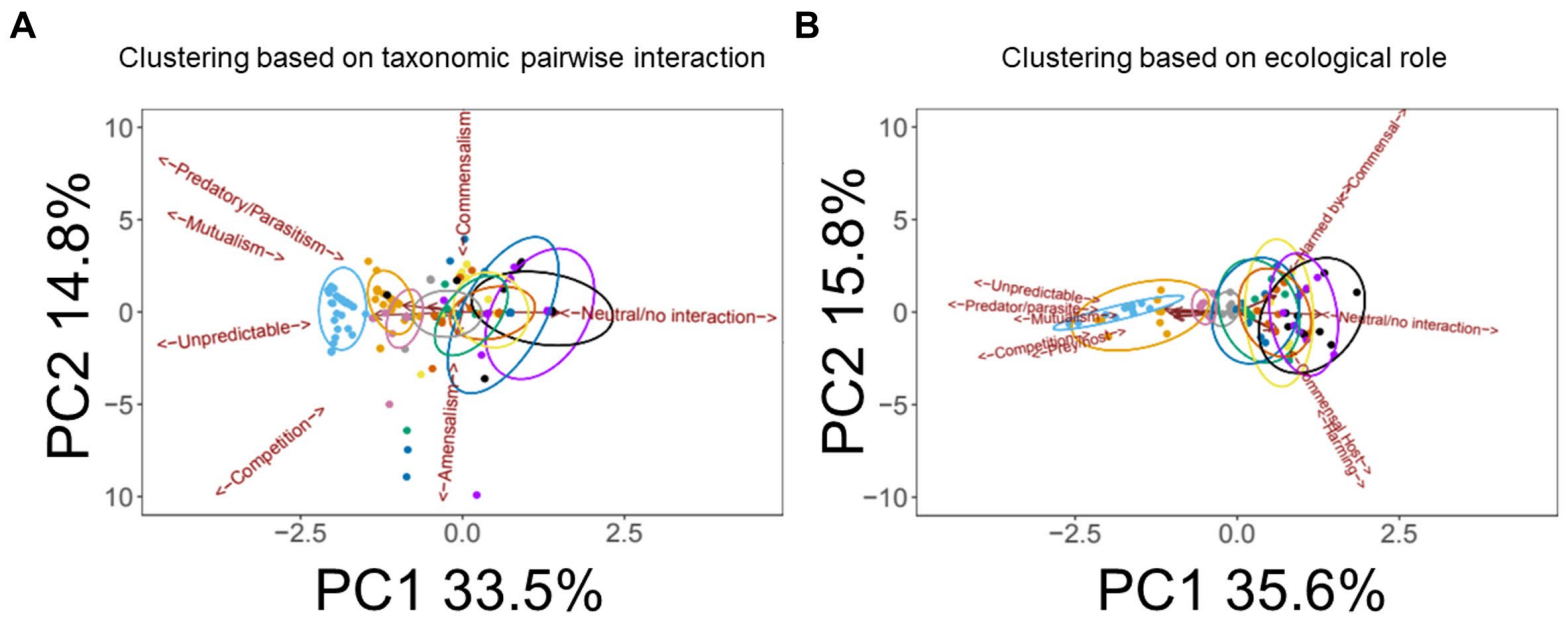
Impact of medications in the “others” category on pairwise taxonomic interactions and ecological role(s). **(A)** Principal Components Analysis (PCA) scores plot showing the impact of medications in the “others” category on pairwise interactions within the wider category “antimicrobials + pancrelipase + others.” Trends associated with different types of ecological interaction (mutualism, commensalism etc.) are indicated. **(B)** PCA scores plot showing the impact of the indicated medications on individual taxonomic categories within the wider category “antimicrobials + pancrelipase + others.” Color key: 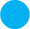 DNase, 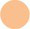 supplementaries, 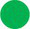 DNase + supplementaries, 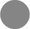 mucolytic agents + supplementaries, 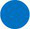 β-agonists + supplementaries, 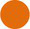 β-agonists + mucolytic agents + supplementaries, 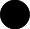 β-agonists + DNase + supplementaries, 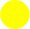 DNase + mucolytic agents + supplementaries, 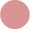 β-agonists + DNase + mucolytic agents, 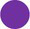 β-agonists + DNase + mucolytic agents + supplementaries. Ellipses indicate 95% confidence intervals.

In the Results outlined above, we note that the “others” category of medication is very broad, incorporating a wealth of interventions, including beta agonists (bronchodilators), mucolytic agents (chemical mucus thinners, not including enzymes) and DNase. Most pwCF routinely receive one or more of these medications (Edmondson and Davies, 2016), with prescriptions >50% in homozygous pwCF ΔF508 (Supplementary Figure S3). Additional interventions in this broad general category include vitamins, proton pump inhibitors, steroids, and antacids – which we collectively denote here as “supplementary treatments” for simplicity. We therefore examined whether we could discriminate the impact of these commonly-used interventions (beta agonists, mucolytic agents, DNase, or “supplementary treatments”), within the category “antimicrobials + pancrelipase + others.” A PCA of the grouping “antimicrobials + pancrelipase + X,” where X is one or more of beta agonists, mucolytic agents, DNase, or supplementary treatments, is shown in Figure 6. The data clearly show, for segregation based either on pairwise interactions between taxa (Figure 6A) or on the ecological role(s) of each taxon (Figure 6B), that DNase treatment has a large impact on inter- and intra-species interactions.

## Discussion

Until now, most previous studies have focused on cataloguing the *CF* airway microbiome and the impact of selected medications on this. However, to our knowledge, very few studies have had the goal of examining how therapeutic interventions influence the interactions between species [exceptions being (Magalhães et al., 2017; O’Brien and Welch, 2019; O’Brien et al., 2021; Ghuneim et al., 2022; Jean-Pierre et al., 2023)], especially on a large (nationwide) dataset. The aim of this work was therefore two-fold. First, we wanted to investigate whether distinct ecological signatures (arising due to inter- and intra-species/taxa interactions) could be identified by analysis of the UK *CF* Registry data using a generalized Lotka-Volterra model. Second, we wanted to examine whether the microbial interactome is affected by the type of medication received by pwCF. Our results indicate that inter- and intra-species ecologies can indeed be inferred from the Registry data, and that certain medications do have an influence on the stability/dynamics of the ecosystem.

Initially, we were uncertain whether application of a gLV approach to the Registry data would yield clear interactome signatures. To understand the basis for this reticence, consider the best-known historical application of the LV model; the analysis of correlated lynx and hare population fluctuations in Canada over the period 1845–1935. The data for those analyses were based on the annual sales of lynx and hare pelts to the Hudson’s Bay Company. Superficially, our approach was similar, being based on annual nationwide reports collected by the *CF* Registry. However, lynx and hare populations ought to be correlated (due to the established predator–prey relationship between these animals). By contrast, there is no reason why species fluctuations in the *CF* Registry data should display this type of synchrony, since any patterns in individual patients may well be “averaged out” in the larger dataset. However, this was not the case, and we were able to identify signatures of inter-species interaction in the data, some of which appeared strongly-linked with certain medications.

For simplicity, we chose to limit our analyses to those pwCF who are homozygous for the ∆F508 mutation. This is the commonest mutant allele in the UK *CF* population (50.4% of the pwCF in the Registry were homozygous for ∆F508, and 88.0% of pwCF carried at least one ∆F508 allele). In addition, the phenotypic defect caused by the ∆F508 allele is partially corrected by the current generation of CFTR modulator therapies, such as Kaftrio. The Registry data relating to the microbiota for pwCF ∆F508 comprised presence/absence records for the identifiable taxa, as well as records of the therapeutic interventions being deployed. To ensure computational feasibility and adequate taxonomic coverage, we focused our analyses on those microbial taxa whose annual prevalence exceeded 5% (abundance). Moreover, we did not further differentiate intra-taxonomic variants (small colony variants, mucoidy etc) in our analyses. However, in spite of this rather “sparse” information, the gLV approach consistently yielded reproducible probabilistic distributions of ecological interactions. Depending on the medication and specific pairwise species/taxa interaction being considered, the simulations revealed signatures consistent with almost all types of ecological interaction (commensalism, amensalism, mutualism etc.). However, it should be noted that our results indicate that these ecological signatures are often fluid, and the organisms in a given pairing can display different ecological interactions within- or between years, and within a given medication group (Supplementary Figure S13).

Perhaps the most notable result we obtained was that treatment with “antimicrobials + pancrelipase + others” affected competition, mutualism, predatory/parasitic and neutral interactions between all of the taxa examined. Since causation cannot be inferred from gLV analyses, we cannot comment further on the possible mechanistic basis for these observations. After antimicrobial agents, pancrelipase was the most highly-prescribed therapeutic intervention among pwCF in the Registry data. Many pwCF exhibit pancreatic insufficiency, leading to impaired digestion of many foodstuffs and consequently, nutritional limitation. The reduced uptake of fats also leads to a reduction in the uptake of fat-soluble vitamins, leading to additional problems. To overcome this, many pwCF are prescribed pancreatic enzyme replacement therapy (PERT), most commonly through treatment with pancrelipase. This mixture of porcine amylase, lipase and protease has been proven to assist in the digestion of polysaccharides, fats and proteins (respectively). There are two possible outcomes of PERT, neither of which is mutually exclusive. First, the therapy could alter the composition of the gut microbiota directly by changing the nutritional environment of the GI tract. Consistent with this, pancrelipase has been shown to alter the gut microbiome in mice (Nishiyama et al., 2018), although it is questionable whether the mouse diet and gut microbiome are a good model for the human. Nevertheless, it is now reasonably well established that the gut microbiota can influence the airway microbiome (the “gut-lung axis” model) (Price and O’Toole, 2021). However, the mechanism(s) by which the gut microbiota influence the lung microbiota remain unclear. Second, pancrelipase could lead to a direct change in the amount and makeup of circulating nutrient, which, in turn, could influence the activity of the lung microbiota. Short chain fatty acids such as acetate, propionate, and butyrate have long been implicated as being particularly relevant in this regard, since they are known to modulate immune responses [reviewed in Deleu et al. (2021)]. However, we note that although treatment with “antimicrobials + pancrelipase + others” elicited a clear ecological signature in all of the orthogonal methods of analysis we applied, treatment with any of these medications on their own, or in dual combinations, did not elicit such a robust response. We also cannot say whether the altered ecology brought about by treatment with “antimicrobials + pancrelipase + others” is beneficial to the cohort – only that it happens.

We also further dissected the possible involvement of individual medications under the “others” heading within the treatment category “antimicrobials + pancrelipase + others.” This revealed that DNase appears to play an important role. DNase is deployed as a mucus thinning agent, and does so by digesting the viscous DNA released through bacterial and host cell lysis. DNA is an important component of the extracellular matrix, and as such, acts as a structural adhesive that holds cells together in biofilm-like assemblages (Das et al., 2013; Campoccia et al., 2021). These assemblages are likely to be polymicrobial, and their disassembly or destabilization (as a consequence of DNase action) may well influence inter-taxon interactions. In addition, DNA is a major structural component of neutrophil NETs (Lachowicz-Scroggins et al., 2019; Sorvillo et al., 2019; Masucci et al., 2020; Szturmowicz and Demkow, 2021), so it’s loss would also be expected to affect microbial interactions through this route too.

One limitation of the current study is that we have only been able to define the influence of medications on a relatively small subset of taxa (reflecting the species that are typically routinely cultured or detected in the clinical laboratory tests). We anticipate that with a more comprehensive and quantitative dataset (obtained from, e.g., 16S/18S rDNA analyses) the outcomes may be more informative. Another limitation is the necessarily rather coarse definition of “treatment categories.” In an ideal world, we would have resolved the treatment options further still, e.g., by separating “antimicrobial agents” into anti-fungal, anti-pseudomonal, anti-staphylococcal etc. However, and in spite of the large size of the dataset employed in this study, the sample sizes for such a finer-grained analysis, as well as the computational power required, were limiting.

In summary, we have interrogated the UK *CF* Registry database using a generalized Lotka-Volterra model to look for evidence of inter- and intra-species ecological interactions. To the best of our knowledge, this is the first time that this approach has been applied to such a large, longitudinal dataset. The data reveal that certain medications – especially those that potentially influence the “gut-lung axis,” or potentially, biofilm formation within the airway mucus - have a distinct impact on the ecology of the airways, and that pairwise interactions between taxa can be fluid over time. [It is worth noting here that the CFTR is also present in the epithelial cells of the GI tract (Merjaneh et al., 2022), so CFTR modulators may also influence the gut physiology.] Current efforts are aimed at investigating whether some of the pairwise species interactions that we observed can be recapitulated for further, mechanistic study, *in vitro*.

## Data availability statement

The original contributions presented in the study are included in the article/Supplementary material, further inquiries can be directed to the corresponding author. Access to the raw data was granted (following peer review) by the UK CF Registry (proposals 425 and 425A). The analysis pipeline (with a command-line interface, for high-performance computers equipped with the SLURM system) is available at https://github.com/ph-u/biLVC; data categorization scripts are available at https://github.com/ph-u/UKCFRegDataSorting.

## Ethics statement

NHS research ethics approval (07/Q0104/2 UK Cystic Fibrosis Registry, AB/AM04/1) has been granted for collection of data into the UK *CF* Registry. Each patient provided written informed consent for data collection and for use of anonymized data in research.

## Author contributions

P-MH and MW conceived the study. MW directed the analysis (biological and clinical aspects). P-MH designed and implemented the analysis (ecological and statistical aspects). P-MH, RN, and MW wrote the manuscript together. All authors contributed to review and editing.

## Funding

This work was supported in part by the NC3Rs and by a *VIA* award from the UK *CF* Trust. RN is recipient of the Benn W. Levy - Vice Chancellor Award SBS DTP studentship.

## Conflict of interest

The authors declare that the research was conducted in the absence of any commercial or financial relationships that could be construed as a potential conflict of interest.

## Publisher’s note

All claims expressed in this article are solely those of the authors and do not necessarily represent those of their affiliated organizations, or those of the publisher, the editors and the reviewers. Any product that may be evaluated in this article, or claim that may be made by its manufacturer, is not guaranteed or endorsed by the publisher.
