# Supplementary material for: Therapeutic interventions alter ecological interactions among cystic fibrosis airway microbiota
Source: Front Microbiol. 2023 May 30;14:1178131. doi: 10.3389/fmicb.2023.1178131 (PMC10265647; doi:10.3389/fmicb.2023.1178131)
Supplement: Supplementary file 1 [file Data_Sheet_1.pdf]

Supplementary information

1. Data cleaning

The data used in this analysis were obtained following an application to the UK CF Registry. The raw data were then curated and standardized into a presence/absence record. The presence/absence data was then filtered by microbiota composition, followed by segregation and coagulation into percentage presence time-series for those pwCF who are homozygous for the ∆F508 mutation. Medical interventions were divided into four categories: CFTR modulators, antimicrobials, pancrelipase and others.

1.1 Raw data

The thirteen-year record consists of three data structures. During 2008-2013, microbiota, and medication histories were summarized on one spreadsheet each year. For 2014 and 2015, non-tuberculous Mycobacteria (NTM) were recorded separately from the main record. From 2016 onward, medication histories were recorded in more detail on multiple spreadsheets each year, along with a separate NTM table and the main record page.

The anonymized individual-level records contained just under 120 000 entries from around 13 000 pwCF across years 2008-2020. Around 95% of entries (n $\simeq$ 113 000) contained a record of the microbiota, and were extracted for downstream curation. Data processing was performed in R v4.1.0 unless stated otherwise.

1.2 Text data curation

Spelling variations and typos were commonly found in the records. We collected unique text and grouped these according to microbiota, medications and “data record headers”. This standardized the record structure. Curation involved automated text correction followed by a manual verification. Automated text correction was carried out using an automated Google search under VPN (virtual private network) mediation [1]. The search automation program was scripted in python3 (v3.9.7) using packages beautifulsoup4 (v4.10.0) and requests (v2.27.1). The search result was manually verified using the NCBI Taxonomy Browser [2] and peer-reviewed medication databases (e.g. [3]).

1.3 Presence/absence standardization

The data were reformatted into a presence/absence structure. For multiple choice records, we accepted a “presence" only when the record clearly indicated so. Any ambiguities were categorized as “absence". These cleaned data were from around 5 000 pwCF across 13 years - a total of around 60 000 data entries comprising 502 microbial and 206 medication categories.

1.4 Microbial categories and coagulation

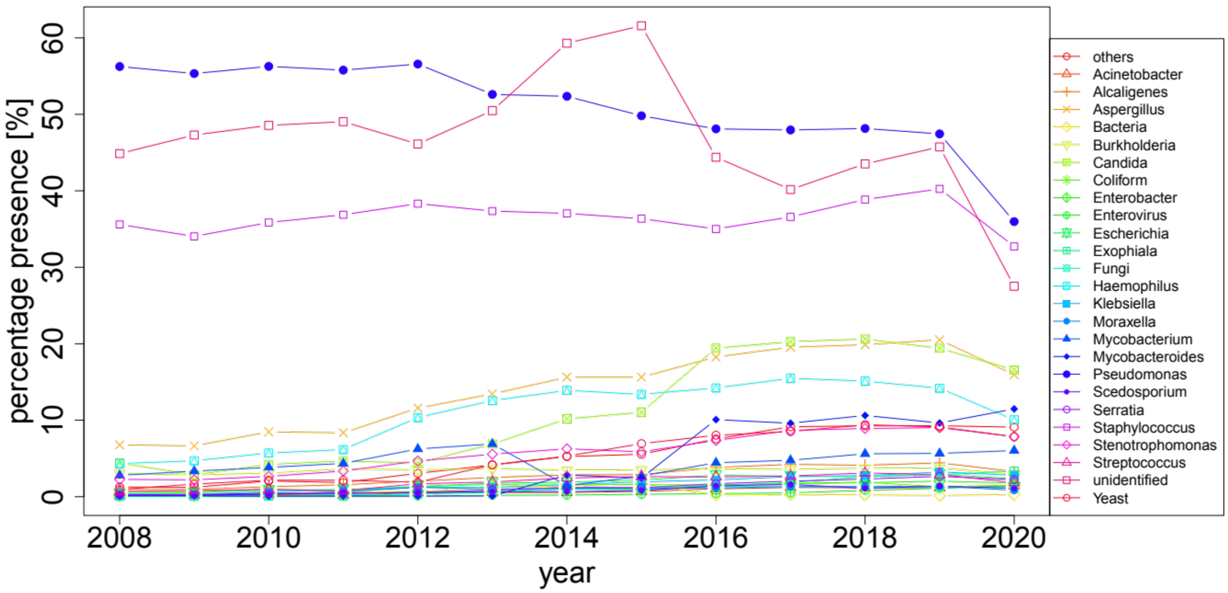

Figure S1: Genera presence trend observed in the UK CF Registry data. Note that an unusual high percentage of “unidentified" were recorded between 2013 and 2015; this peak is likely the cause of the dropped generalized Lotka-Volterra simulation success rate on many medication groups.

The Registry data recorded 502 distinct microbial categories. A time series showing the top 25 most frequently recorded taxa is shown in **Figure S1**. We simplified the 502 categories by coagulating these into 184 genera categories (**Figure S2**). We then applied a 5% presence threshold on the 184 genera categories across all years (2008-2020). The nine microbial categories (e.g. “*Aspergillus*", “*Candida*", “*Haemophilus*" etc) outlined in the **Methods** section were the only ones present in ≥5% of the data entries; the remaining

174 genera were coagulated under “others". Descriptions of each individual category and the list of genera included in “others" were outlined in **section 6**.

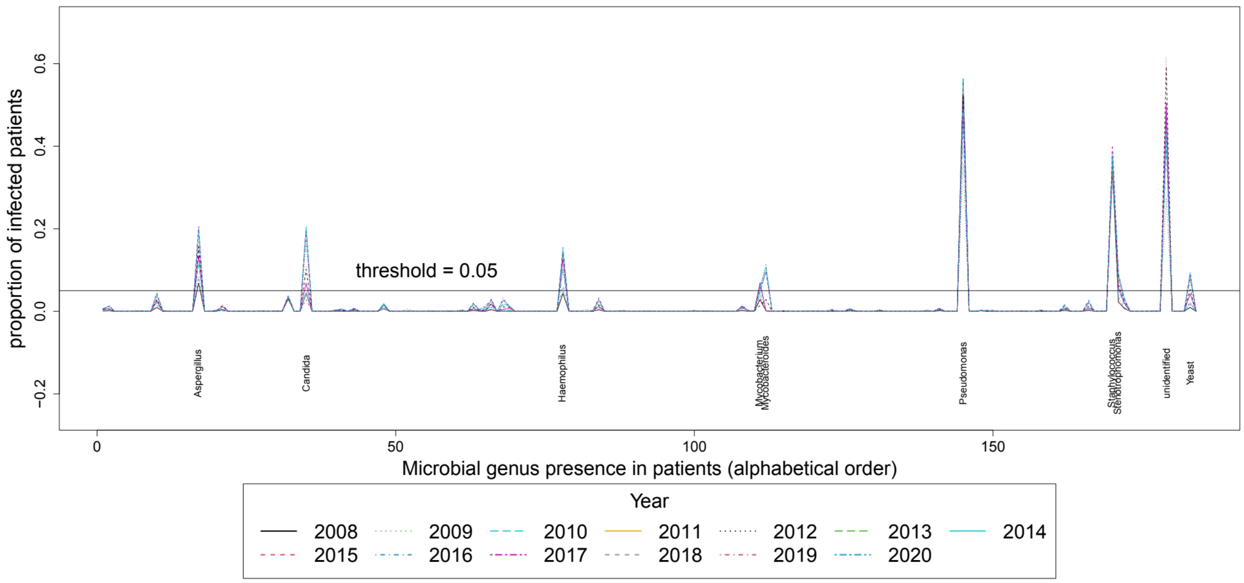

Figure S2: Microbial genera presence ratio among sampled people with cystic fibrosis (pwCF). Note that the genera categories listed had at least one year with presence above the 5% sample threshold. There were 184 categories in total, and only 10 categories had passed the 5% threshold filter being an individual category in the Bayesian Inference adaptive Markov Chain Monte Carlo analysis.

1.5 Medication categories coagulation

Prescriptions made across the 13-year duration were diverse, although as expected, some interventions were more commonly-deployed than others. Some interventions were essentially universally used, whereas others were used by only one or a few pwCF. Given the computational limitations of our approach (even using the Cambridge High

Performance Computing Cluster), we therefore coagulated the therapeutic treatments into 206 functional categories (e.g. “CFTR modulators”, “antimicrobials”, “pancrelipase”, “corticosteroid” etc). To maximize the sample size in each medication grouping and the relevance to CF, we selected “CFTR modulators” and the top two most-used medications (“antimicrobials” and “pancrelipase”) as individual categories; then coagulated the rest into “others”. The 206 medication categories were listed in **section 7**.

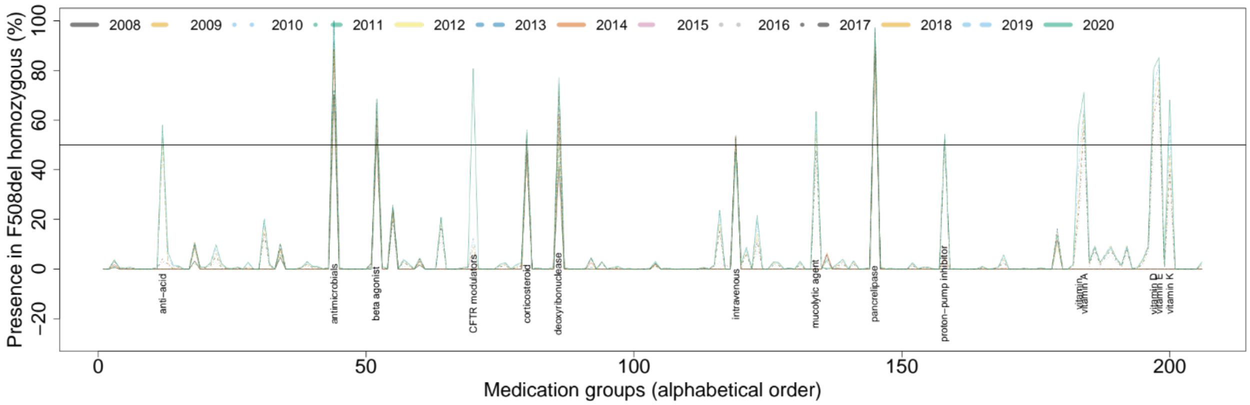

Figure S3: Medication use (graphed by relative frequency of prescription). There were 206 categories in total.

1.6 Time-series segregation

Standardized data entries were further filtered based on the CF allele carried by the individual and grouped by medication prescribed in the recording year. Around 5 000 individuals (50% out of approximately 13 000 catalogued pwCF) were homozygous for the ΔF508 mutation, and the time-stamped entries for these individuals were extracted. These extracted entries were then segregated into eight medication groupings (e.g.

“CFTR modulators", “antimicrobials", “antimicrobials + pancrelipase" etc), as outlined in the **Methods** section. The resulting 8 time-series on the yearly percentage presence of each of the 10 microbial categories were then coagulated. Each time-series was segregated into three-year time windows for analysis using the generalized Lotka-Volterra equation; a total of 69 three-year time windows were segregated.

2. The generalized Lotka-Volterra equation

$$\dot{x}_i = \frac{dx_i}{dt} = x_i\left(r_i + \sum_{j=1}^{n} \alpha_{ij}\, x_j\right) \qquad \textbf{(1)}$$

where $\dot{x}_i$ (or $\frac{dx_i}{dt}$) is the maximum growth rate of population i per unit volume (unit: N $V^{-1}t^{-1}$), r_i is its cellular maximum growth rate (unit: t^{-1}), x_i and x_j are the population i (per unit volume) and its interacting population j (per unit volume) at that time-step (unit: NV^{-1}), α_{ij} is the cellular interaction rate describing how i is influenced by j at the time-step (unit: $N^{-1}t^{-1}$; positive = synergistic, negative = antagonistic).

3. Priors

From the generalized Lotka-Volterra equation (**Equation 1**), we formulated boundary conditions (priors) for estimations of two types of parameters - maximum growth rates (r_i) and interaction coefficients (α_{ij}) as outlined in the **Methods** section.

Rough estimates of growth rates were obtained from statistical linear analyses (between two consecutive years) of each microbial time-series. The range of slope values were set

as the rough boundaries and the means of these slope values were set as the starting values for downstream refinement. With the agnostic boundaries of interaction coefficients (-2 to +2) and using “0" as the starting values, each time-series was optimized by the Nelder-Mead method. The mathematical integrator selection was automatic, choosing between the "RK4" or "Euler" algorithm. Refinements were performed using R packages “FME" (v1.3.6.2) [4], “deSolve" (v1.30) [5], “rootSolve" (v1.8.2.3) [6, 7] and “coda" (v0.19.4) [8]. The refined parameter values were used as starting values for the Bayesian Inference adaptive Markov Chain Monte Carlo (aMCMC) simulations. Prior boundaries were modified by the difference between the initial boundary and refined values only if the refined values were out of the range of rough estimations.

4. Bayesian Inference adaptive Markov Chain Monte Carlo

Guided by the time window data, we ran 7 seed-mediated aMCMC chains using the same set of priors under the Bayesian Inference framework. Each aMCMC chain had a length of 500 000 simulations and output 500 best-fit parameter combinations (the top 0.1%). Covariance was updated after every 50 simulations with zero burn-in length. The lack of burn-in length allowed the system to capture sub-optimized parameter value combinations that could also fit the time window data; this step abandoned assuming the optimized value combination being the peak of a unimodal distribution. The R packages used were the same as the prior-construction step listed in the previous section.

5. Ecological relationships and patterns

Output parameter combinations were paired by categories using the logic outlined in **Table S1**. The ecological interactions (outlined in **Table S2**) were collected for two independent variables: therapeutic interventions (**Figure S3**) and microbial taxonomic category (**Figure S2**). This allowed us to assess "therapy *versus* ecological interactions" and "taxonomy *versus* ecological interactions". We summarized 440 taxonomic/species pairs (i.e., 55 pairs × 8 therapies; the 55 pairs coming from 45 inter-species pairs + 10 intra-species pairs) along all time-windows, and 80 "taxonomic tags" (i.e., 10 "taxa/species" x 8 therapies; each "species" summarizing 9 inter-species + 1 intra-species interaction). To visualize the effect of the independent variables, we used Principal Components Analysis (PCA), Partial Least Squares-Discriminant Analysis (PLS-DA; supervised by the independent variable of interest; R package mixOmics v6.18.1 [9]) and radar plots (R package fmsb v0.7.5 [10]). To understand the combined effect of therapies and taxonomy, we employed a hierarchical cluster analysis (HCA) with Euclidean dissimilarity distances and centroid clustering method on the 80 taxonomic tags.

Table S1: A sample triple species interaction matrix. Note that the matrix represents how the “column" population affects the colonization dynamics of the “row" population.

	p1	p2	p3
p1	[1,1]	[1,2]	[1,3]
p2	[2,1]	[2,2]	[2,3]
p3	[3,1]	[3,2]	[3,3]

Table S2: Summary of definitions on ecological interactions for this study. Note that there are nine ecological roles from five types of ecological relationships.

Microbial effect		Ecological	Ecology	Pairwise interaction	
A on B	B on A	role of microbe A	type	Intra-species	Inter-species
+	+	Mutualism	Mutualism	Yes	Yes
+	0	Commensal host	Commensalism		Yes
+	-	Prey/host	Predatory/Parasitism		Yes
0	+	Commensal	Commensalism		Yes
0	0	Neutral/no interaction	Neutral	Yes	Yes
0	-	Harmed by B	Amensalism		Yes
-	+	Predator/parasite	Predatory/Parasitism		Yes
-	0	Harming B	Amensalism		Yes
-	-	Competition	Competition	Yes	Yes

6. Microbial taxonomies and brief descriptors

“***Aspergillus***" A genus of fungi that are increasingly associated with poor outcomes in CF (e.g. [11–13]). *Aspergillus* can cause allergic bronchopulmonary aspergillosis (ABPA), which is an allergic response towards the hyphae of this fungus [14]. *Aspergillus fumigatus*, an observed CF airway microbe, can produce aflatoxins [15–17].

“***Candida***" *Candida* can cause allergic bronchopulmonary candidiasis (ABPC) [12, 18-19]; an allergic response towards the presence of *Candida* species. Patients with ABPC

show a lower level of serum antibody IgE because this antibody is the main defense against the invader [18].

"***Haemophilus***" (Winslow et al. 1917) is a genus category (in bacteria) in the CF airways (e.g. [11, 20, 21]). Non-typeable *Haemophilus influenzae* (NTHi) can cause a pro-inflammatory chronic infection, which increases T-cell burden and recruitment of neutrophils [22, 23]; the species is also responsible for early childhood infections [24]. *H. influenzae* and *H. parainfluenzae* have mosaic genomes that can be reservoirs of antimicrobial resistance (AMR) genes [25].

"***Mycobacteria***" is a combined category including "*Mycobacterium*" [26–28] and "*Mycobacteroides*" [29, 30]. The genus includes several opportunistic pathogens which may lead to chronic/lethal infection [31]. *Mycobacteroides abscessus* is a widespread environmental opportunistic pathogen [32] associated with inflammation [33, 34]. The *M. abscessus* complex [35] is of increasing concern due to their diverse collection of antibiotic resistance mechanisms [33, 35].

"***Pseudomonas***" [*aeruginosa*] is perhaps the most commonly-encountered CF-associated pathogen [26, 36]. Known for its broad AMR profile, biofilm-forming ability, and secreted virulence factors.

"***Staphylococcus***" [*aureus*] is another commonly-encountered CF-associated pathogen [37, 38]. The species is responsible for early childhood infections [24].

“***Stenotrophomonas***" [*maltophilia*] an emerging opportunistic pathogen in CF airways [39, 40], known for its possible role(s) in acute pulmonary exacerbation events, biofilm formation and AMR profile [41, 42].

“**Yeast**" Includes all other non-candida/aspergillus fungal pathogens.

“**Unidentified**" microbes are commonly recorded in the CF Registry data, and include tags such as “normal flora" or “normal mouth flora".

Another common (but distinct, in terms of the Registry records) category was “**others**" This group was present in < 5% of pwCF and was often associated with the following tags.

- **Bacteria/Eubacteria** (111 sub-categories): *Achromobacter, Acinetobacter, Actinomyces, Advenella, Aerococcus, Aeromonas, Aggregatibacter, Agrobacterium, Alcaligenes, Anaerobiospirillum, Arthrobacter, Atlantibacter*, Bacillus, Bacteria, *Bacteroides, Bergeyella, Bordetella, Brevibacterium, Brevundimonas, Brucella, Burkholderia, Buttiauxella, Campylobacter, Capnocytophaga, Cedecea, Chlamydia, Chromobacterium, Chryseobacterium, Citrobacter, Clostridium*, Cocci, Coliform, *Comamonas, Corynebacterium, Cronobacter, Cupriavidus, Delftia, Eikenella, Elizabethkingia, Empedobacter, Enterobacter, Enterococcus, Escherichia, Ewingella, Fusobacterium, Gemella, Gluconacetobacter, Gordonia, Granulicatella, Hafnia, Helicobacter, Herbaspirillum, Inquilinus, Klebsiella, Kluyvera, Kocuria, Leclercia, Lelliottia, Leuconostoc, Lysinibacillus, Lysobacter, Mannheimia, Massilia, Metabacillus,*

Microbacterium, Micrococcus, *Mixta, Moellerella, Moraxella, Morganella (include species Morganella morganii; a puffball fungus shared this genus name but is unrelated to CF airway), Mycolicibacillus, Mycolicibacter, Mycolicibacterium, Mycoplasma, Myroides, Neisseria, Nocardia, Ochrobactrum, Oligella, Paenibacillus, Pandoraea, Pantoea, Pasteurella, Peribacillus, Phyllobacterium, Pluralibacter, Prevotella, Proteus, Providencia, Pseudescherichia, Pseudochrobactrum, Rahnella, Ralstonia, Raoultella, Rhizobium, Rhodococcus, Rodentibacter, Roseomonas, Rothia, Salmonella, Schaalia, Segniliparus, Serratia, Shewanella, Sphingobacterium, Sphingomonas, Streptococcus, Tsukamurella, Variovorax, Wautersiella, Yersinia*

- **Fungi** (48 sub-categories): *Acremonium, Alternaria, Apiotrichum, Arthrographis, Aureobasidium, Bjerkandera, Blastobotrys, Chrysosporium, Cladosporium, Clavispora, Coniochaeta, Cordyceps, Cryptococcus, Cutaneotrichosporon, Exophiala*, Fungi, *Fusarium, Geosmithia, Geotrichum, Lichtheimia, Lodderomyces, Lomentospora, Madurella, Malassezia, Meyerozyma, Mucor, Naganishia, Paecilomyces, Papiliotrema, Penicillium, Phanerochaete, Phoma, Pneumocystis, Purpureocillium, Rasamsonia, Rhizomucor, Rhizopus, Rhodotorula, Saccharomyces, Sarocladium, Scedosporium, Scopulariopsis, Talaromyces, Tilletiopsis, Trichoderma, Trichophyton, Trichosporon, Yarrowia*
- **Viruses** (15 sub-categories): *Alphainfluenzavirus, Betainfluenzavirus, Bocaparvovirus, Cardiovirus, Coronavirus, Cytomegalovirus, Enterovirus,*

Influenza, Lymphocryptovirus, Mastadenovirus, Metapneumovirus, Orthorubulavirus, Parechovirus, Picornaviridae, Pneumoviridae

7. Medication categories

The 206 medications analyzed in **Figure S3** are listed individually below.

5-HT4 receptor agonist
Acetylcholinesterase inhibitor
Acetylcysteine
ADHD drug
Adrenaline
Alpha blocker
Alpha-2 agonist
Amiloride
Angiogenesis inhibitor
Angiotensin converting enzyme inhibitor
Angiotensin receptor blockers
Anti-acid
Anti-allergic
Anti-androgen
Anti-anginal
Anti-arrhythmics
Anti-arrhythmicss
Anti-cholinergic
Anti-coagulant
Anti-convulsant
Anti-dementia

Female hormone
Furosemide
Geroprotector
Growth hormone
Growth hormone inhibiting hormone
Guanylate cyclase C agonist
HCN channel blocker
Heavy metal cleaning
Hydrocortisone
Immunosuppressive
Immunotherapy
Inosine monophosphate dehydrogenase inhibitor
Insulin
Interferon
Interleukin-1 inhibitors
Intravenous
Iodine
Iron
Lactic acid
Laxative
Lipoic acid

Anti-depressant
Anti-diabetic
Anti-diarrhea
Anti-diuretic
Anti-emetic
Anti-female hormone
Anti-fibrinolytic
Anti-foaming
Anti-hemorrhagic
Anti-histamine
Anti-hypertensive
Anti-hypotensive
Anti-leukotrienes
Anti-oxidant
Anti-parkinsonian
Anti-perspirant
Anti-psychotic
Anti-rejection
Anti-seizure
Anti-spasmodics
Anti-vertigo
Antibody
Antimicrobials
Anxiolytic
Appetite stimulant

Loratadine
Magnesium
Manganese
Mannitol
Mast cell stabilizer
Mesalamine
Mineral
Mirabegron
Mood stabilizer
Mucolytic agent
Muscarinic antagonist
No medication
Non-benzodiazepine hypnotics
Nonsteroidal anti-inflammatory drug
Nutrient
Ophthalmic
Opioid
Opioid receptor antagonist
Organic nitrates
Pain-relief
Pancrelipase
Parathyroid hormone
Pegloticase
Peppermint oil
Phosphate reduction

Apremilast
Aromatase inhibitor
Ataluren
Azelaic acid
BAAC
Beta agonist
Beta blocker
Betamethasone
Bile acid
Bile acid sequestrant
Bisphosphonate
Blood homeostasis
Blood thinner
Bronchodilator
Buccolam
Buprenorphine
Butanediol
Calcium
Carbonic anhydrase blocker
Carbonic anhydrase inhibitor
Carboxymethylcellulose
Carotene
Carotenoids
CFTR modulators
Chemotherapy

Phosphodiesterase inhibitor
Piperidine
Potassium
Prebiotics
Probiotics
Propulsive
Proteases
Protectant
Proton-pump inhibitor
Rapamycin inhibitor
Red blood cell production
Reductase blocker
Retinoid
Rheology modifier
Sex hormone suppress
Sleeping pill
Smoking cessation aids
Sodium
Statin
Steroid
Sulfamoylbenzamide
Synthetic saliva
Tacrolimus
Testosterone
Thyroid peroxidase inhibitor

Chlorhexidine
Cholesterol-absorption inhibitors
Cholesterol-lowering agent
Choline
Chromium
Colchicine
Contraceptive
Copper
Corticosteroid
Curcuminoid
Cycloheptathiophene
Cysteamine
Decarboxylase inhibitor
Decongestant
Deoxyribonuclease
Depressant
Desmopressin
Dimethyl fumarate
Dimeticone
Disease modifying anti-rheumatic drug
Diuretics
Dopamine agonist
Dopamine antagonist
Dutasteride
Emollient

Trimeprazine
Triptan
Triptorelin
Tumor necrosis factor blocker
Unknown
Urea
Vasoconstrictor
Vasodilator
Vitamin
Vitamin A
Vitamin B
Vitamin B1
Vitamin B12
Vitamin B2
Vitamin B3
Vitamin B4
Vitamin B5
Vitamin B6
Vitamin B7
Vitamin B8
Vitamin B9
Vitamin C
Vitamin D
Vitamin E
Vitamin H

Enterokinetic

Enzyme

Erectile dysfunction therapy

Erythropoiesis stimulating agents

Estrogen receptor modulator

Etoricoxib

Eye drop

Vitamin K

Vitamin P

Wakefulness promoting agent

Weight reduction

White blood cell production

Xanthine oxidase inhibitors

Zinc

8. Graphical results

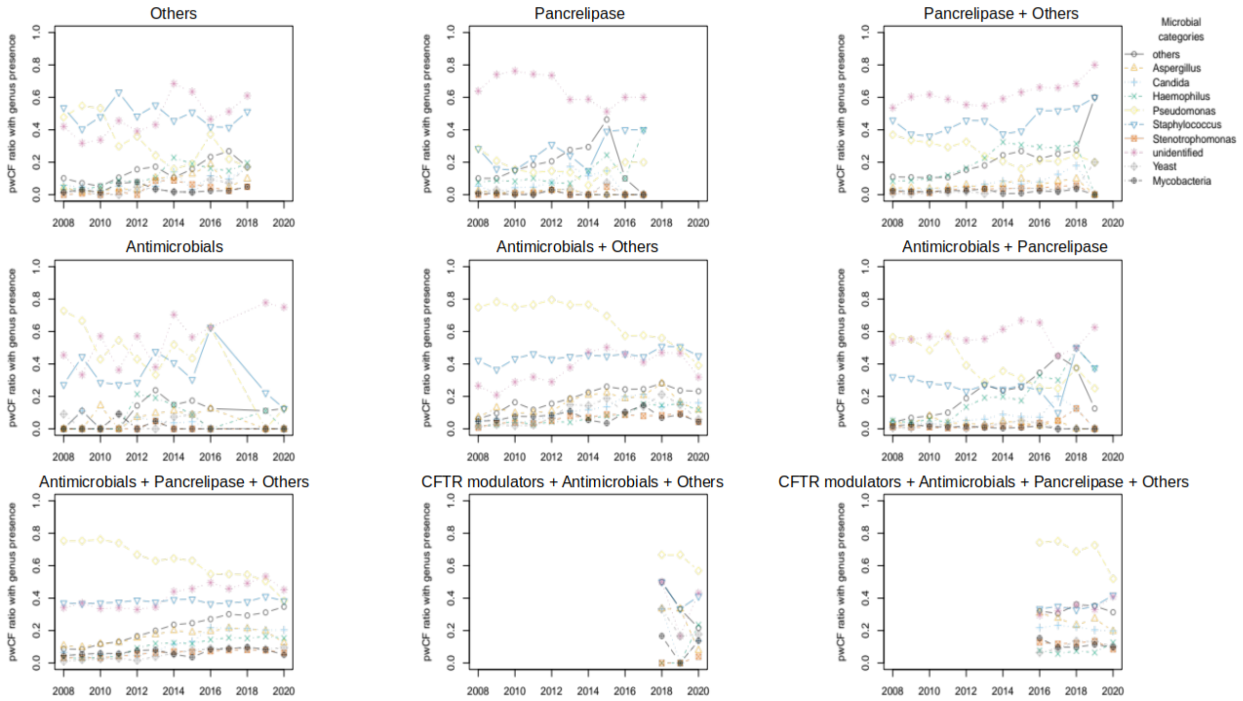

Figure S4: Overview of the data for all medication groups qualifying under the threshold of "each year at least five participants" and "overall time-series at least 30 entries". Note that the group "CFTR modulators + antimicrobials + others" formed a valid time-series but could not be modelled using the gLV. This group was therefore eliminated from downstream analyses.

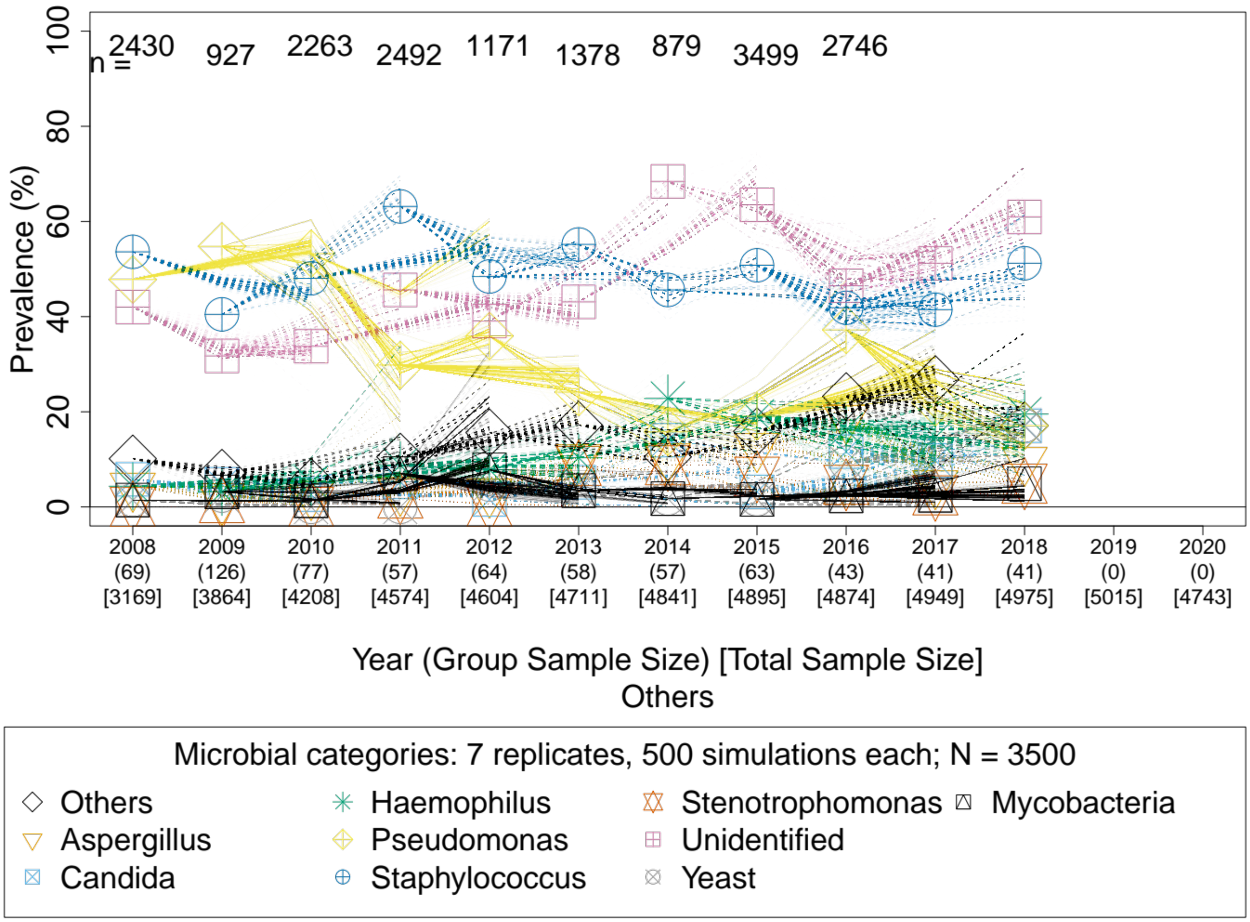

Figure S5: Overview of the top best-fit simulations on data for the medication group “others". "N" is the total number of simulation output (the top 0.1%; 500 top-fit simulations x 7 aMCMC chains). Within these 3500 simulations, we employed a tolerance limit of 25% of any given data point. The number of simulations matching all the data within the time-window is denoted by "n" in the body of the Figure. The n:N ratio represents the

fraction of simulations explainable by the gLV model, symbolizing the stability of ecological interactions within that time window.

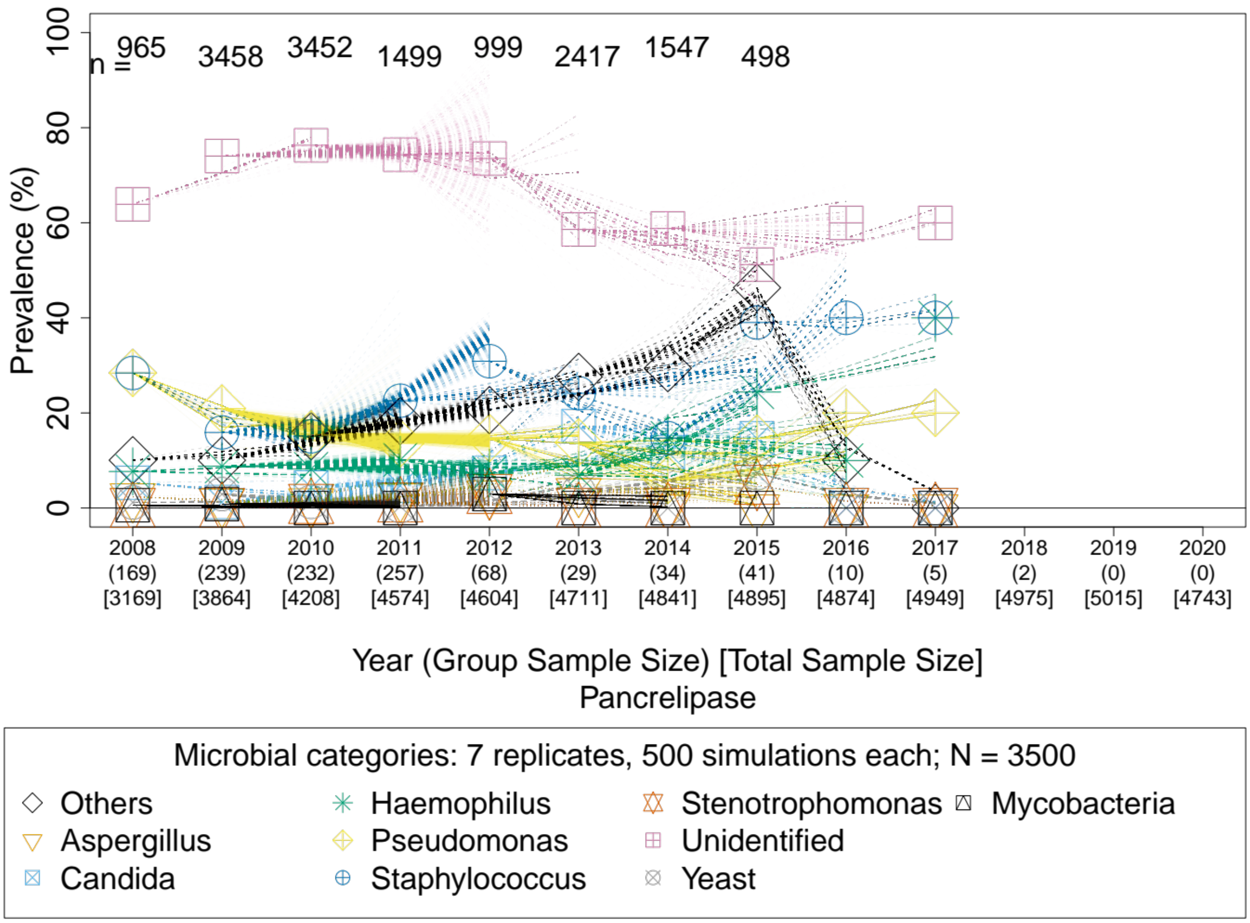

Figure S6: Overview of the top best-fit simulations on data for medication group “pancrelipase". See **Figure S5** for the meaning of N and n in the Figure.

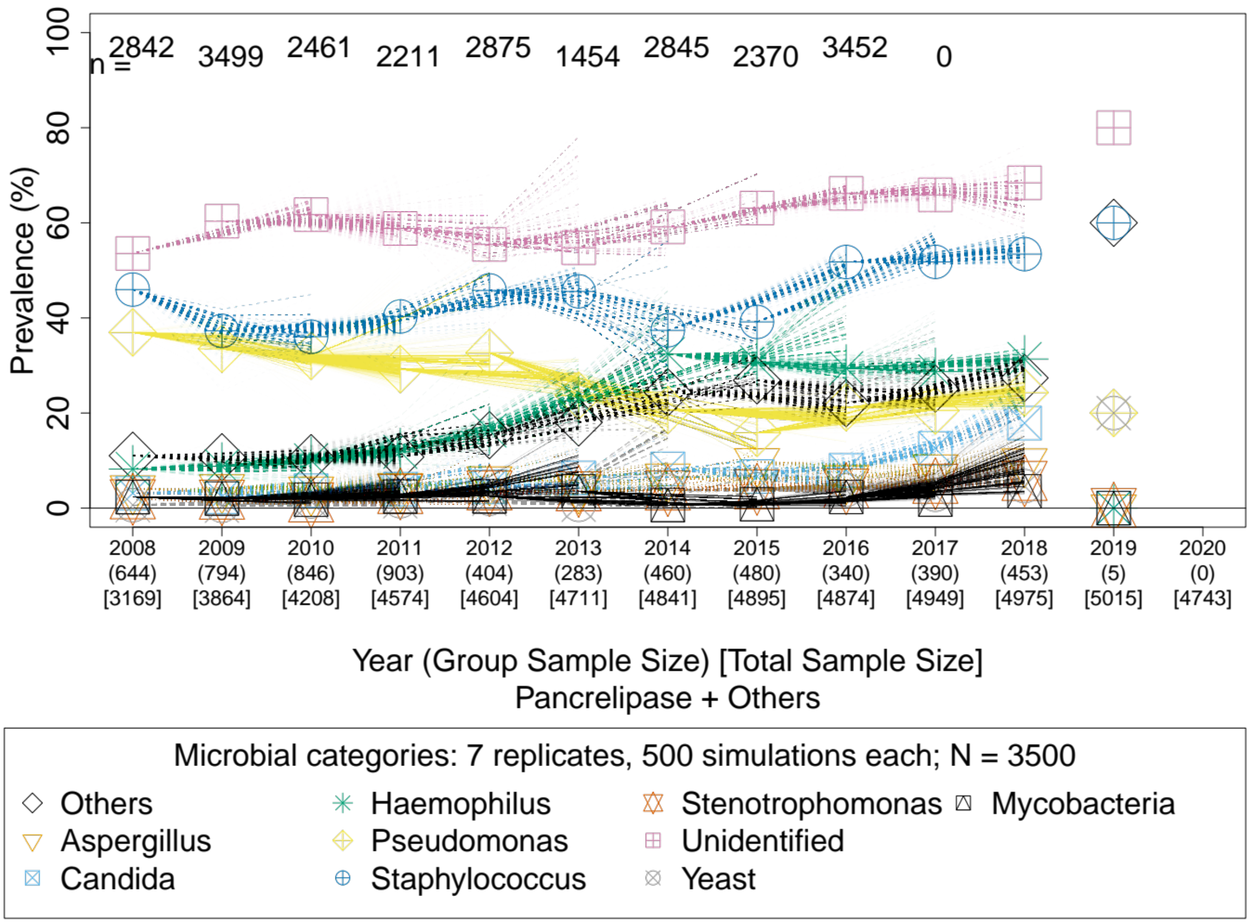

Figure S7: Overview of the top best-fit simulations on data for medication group “pancrelipase + others". See **Figure S5** for the meaning of N and n in the Figure.

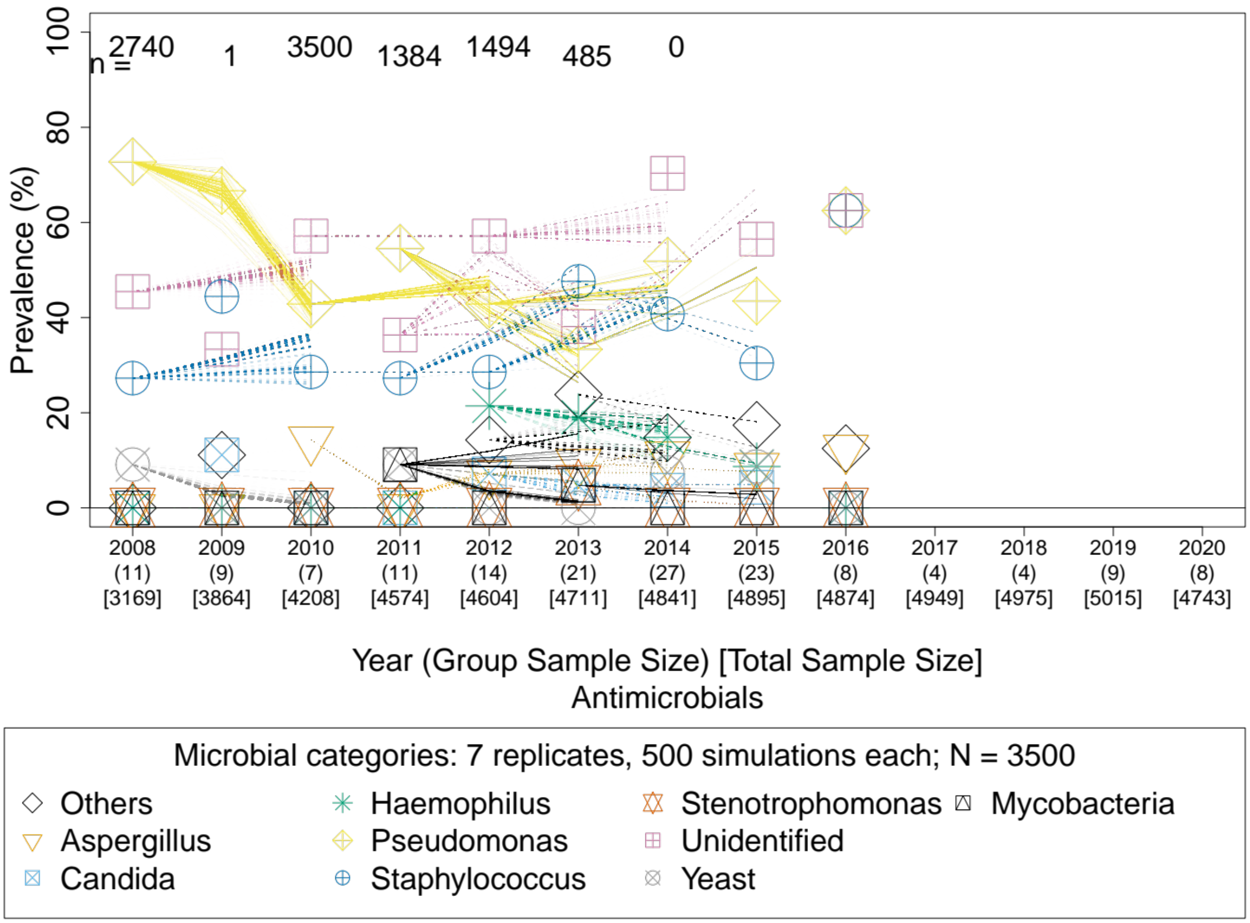

Figure S8: Overview of the top best-fit simulations on data for medication group "antimicrobials". See **Figure S5** for the meaning of N and n in the Figure.

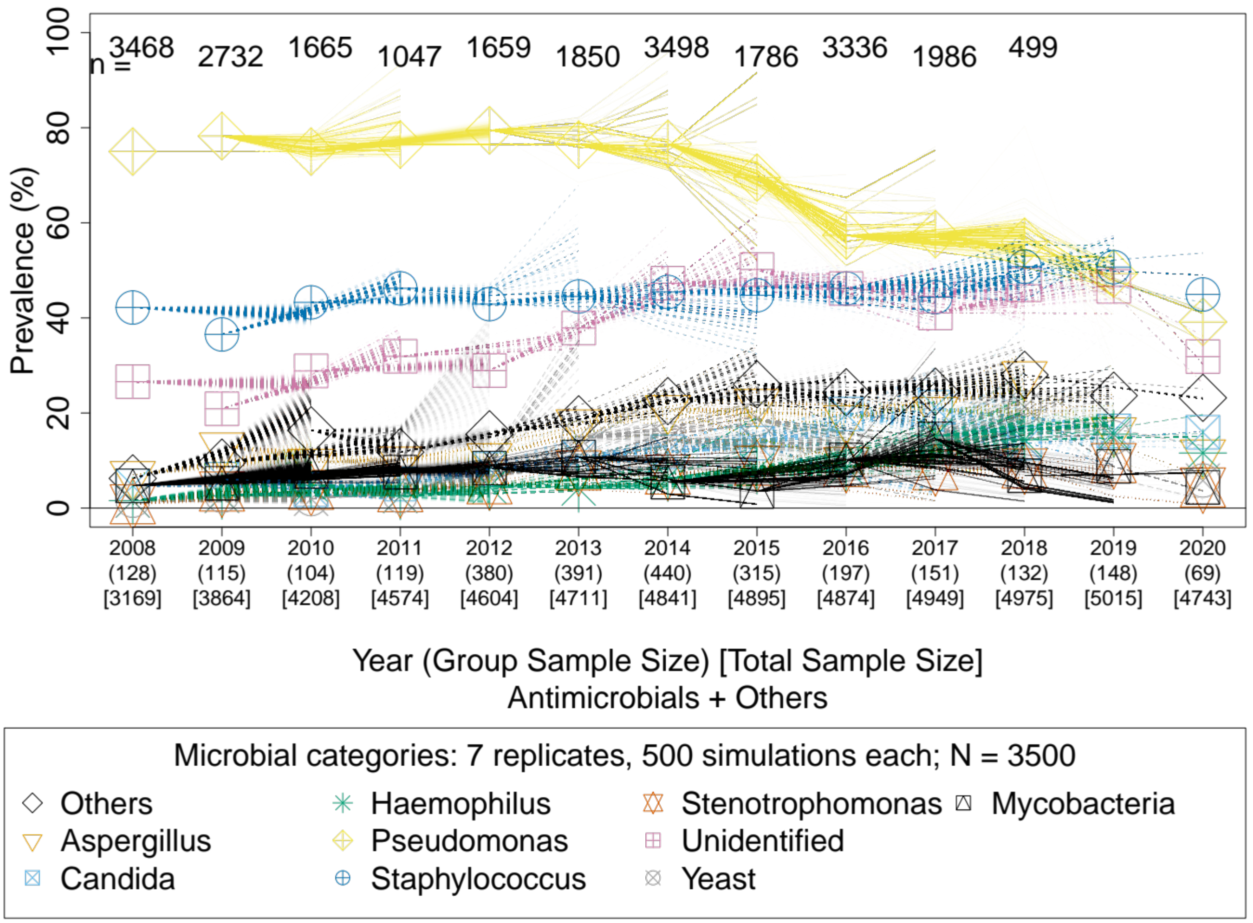

Figure S9: Overview of the top best-fit simulations on data for medication group "antimicrobials + others". See **Figure S5** for the meaning of N and n in the Figure.

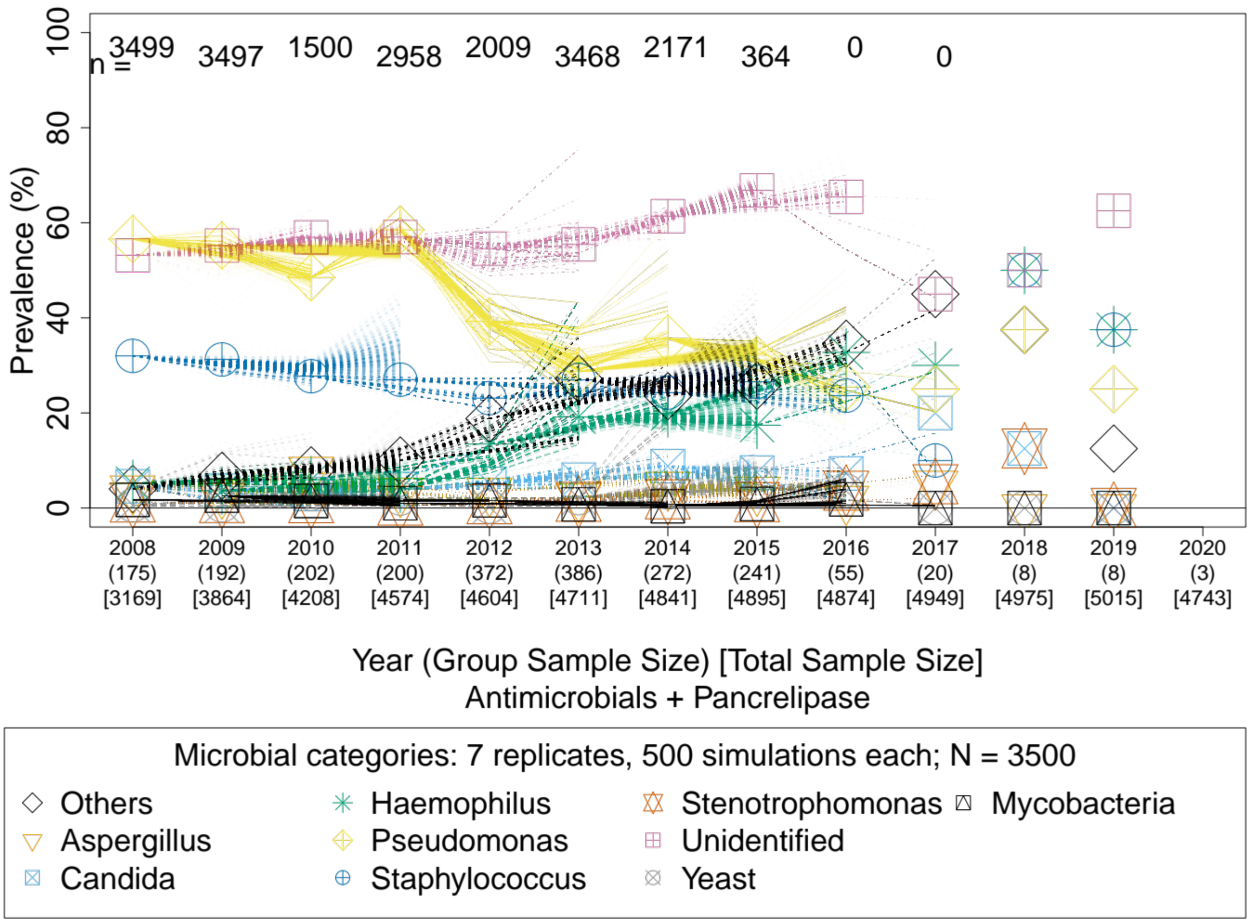

Figure S10: Overview of the top best-fit simulations on data for medication group “antimicrobials + pancrelipase". See **Figure S5** for the meaning of N and n in the Figure.

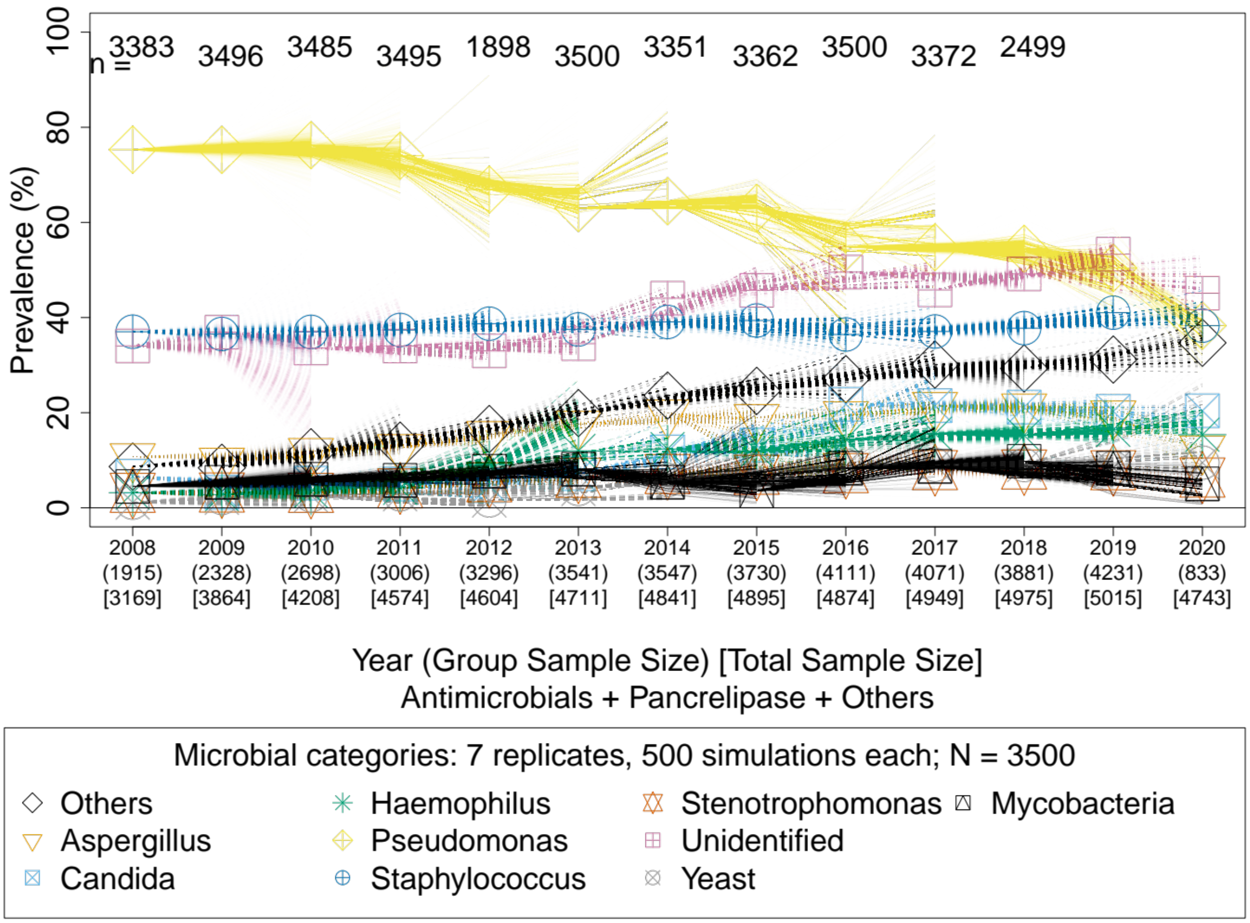

Figure S11: Overview of the top best-fit simulations on data for medication group "antimicrobials + pancrelipase + others". See **Figure S5** for the meaning of N and n in the Figure.

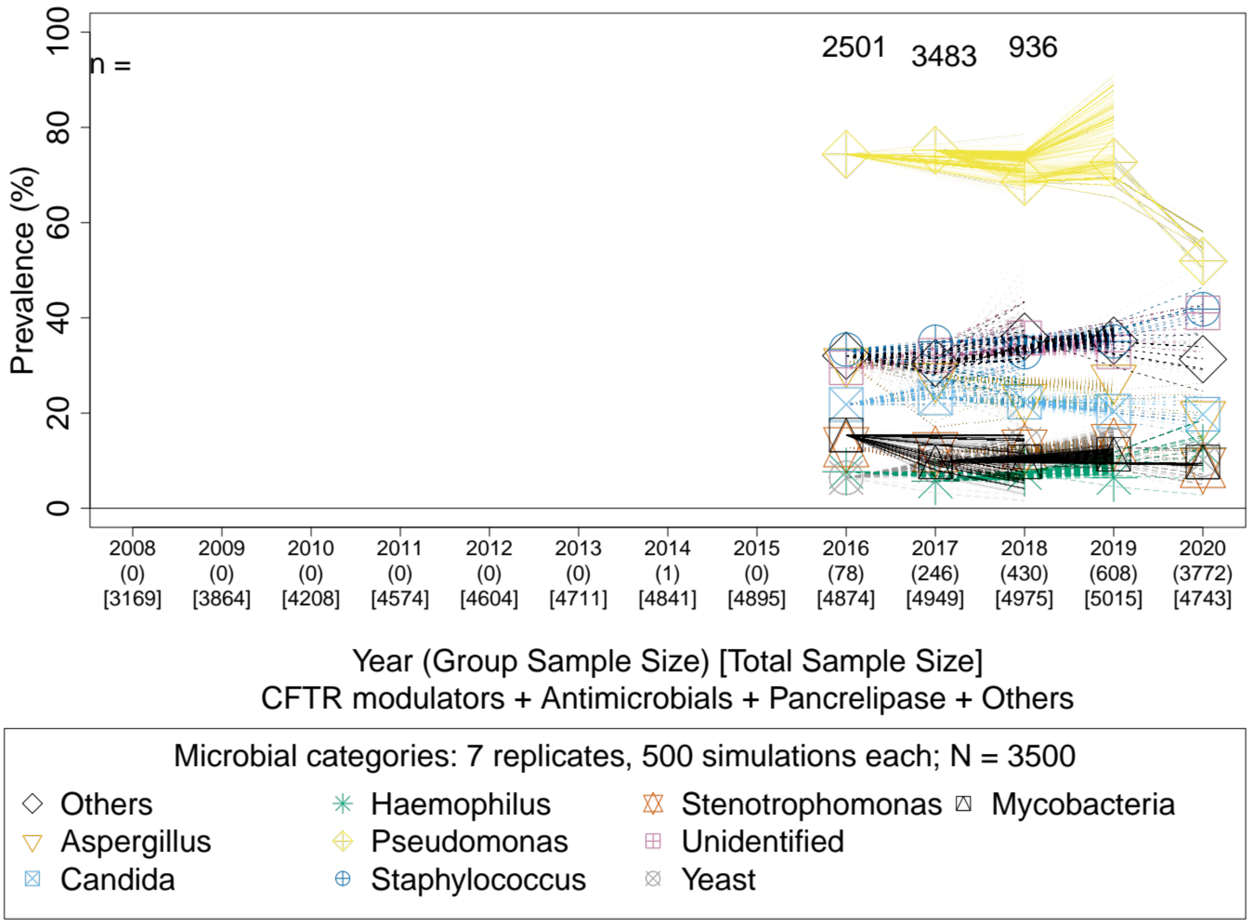

Figure S12: Overview of the top best-fit simulations on data for medication group “CFTR modulators + antimicrobials + pancrelipase + others". See **Figure S5** for the meaning of N and n in the Figure.

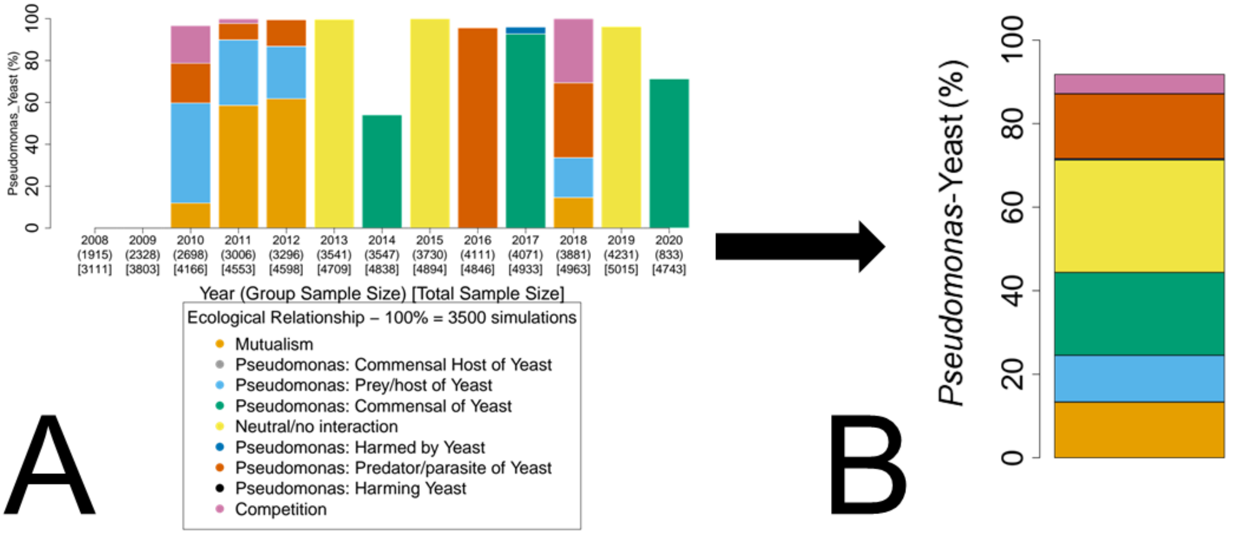

Figure S13: Example of ecological interactions over a rolling three-year time window and their summary into a single data entry. (**A**) Shows the distribution of pairwise interactions between *Pseudomonas* and Yeast over a rolling three-year time window in pwCF ΔF508 treated with "antimicrobial agents + pancrelipase + others". Note that the three-year duration window means that the data for 2008-2009 are presented in the reporting year "2010". Each bar summarizes the ecological interactions derived from 7 MCMC chains of 500 best-fit simulations each (i.e., 3 500 simulations in all). (**B**) The data in (**A**) were combined and condensed to yield a single summary bar representing all of the inter-taxonomic interactions over the period 2008-2020. Note that the final cumulative % on the ordinate in (**B**) is <100%. This is because in any given year, the ecosystem is not perfectly predictable; note that most of the bars for an individual year in (**A**) do not sum to 100%, for example.

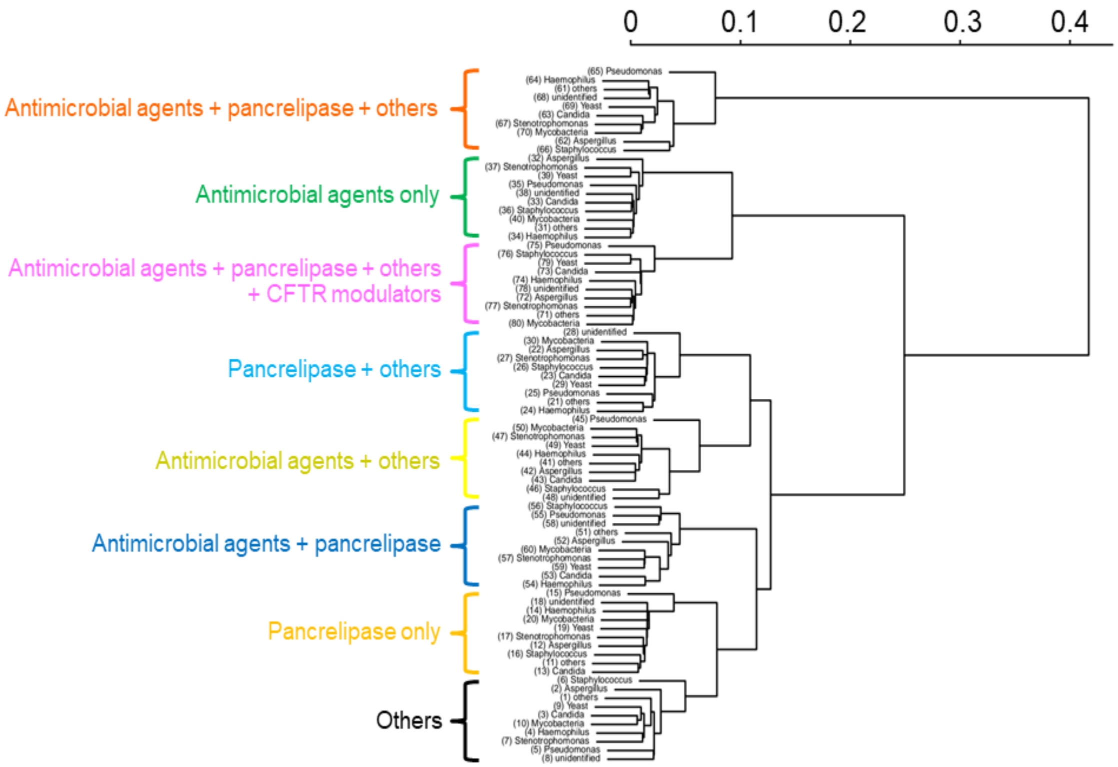

Figure S14: Hierarchical cluster analysis showing the taxonomic tags clustered according to their ecological role(s). The data were segregated using Euclidian distances and clustered by centroids. The data segregated neatly into categories defined by therapeutic intervention. The color coding for the therapeutic interventions is the same as that in **Figure 3**.

Figure S15: Cumulative impact of treatment with “antimicrobials + pancrelipase + others (med)” on the ecological role(s) of the indicated taxa.

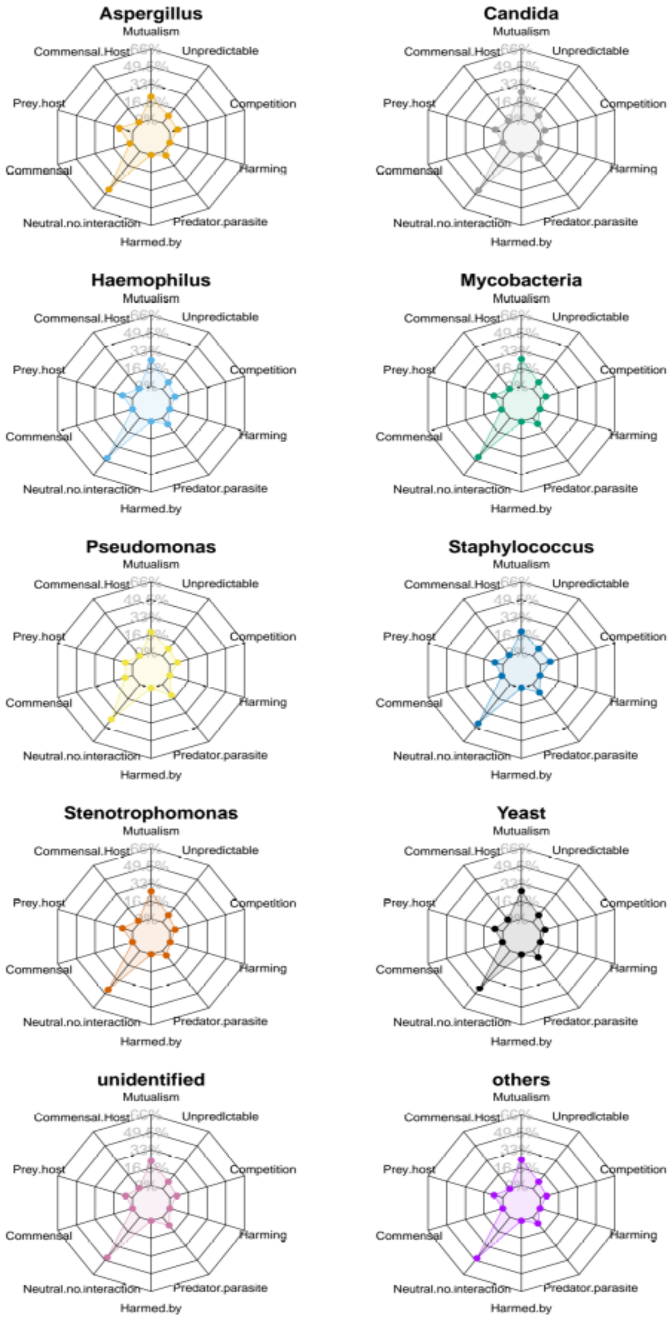

SI References

1. Nobori D. Design and implementation of SoftEther VPN. 2013. Master's thesis, Department of Computer Science, Graduate School of Systems; Information Engineering, University of Tsukuba, Japan.

2. Schoch CL, Ciufo S, Domrachev M, Hotton CL, Kannan S, Khovanskaya R, *et al*. NCBI taxonomy: A comprehensive update on curation, resources and tools. Database. 2020; 2020: 1-21; https://doi.org/10.1093/database/baaa062

3. Wishart DS, Knox C, Guo AC, Shrivastava S, Hassanali M, Stothard P, *et al*. DrugBank: A comprehensive resource for *in silico* drug discovery and exploration. Nucleic Acids Res. 2006; 34: D668–D672; https://doi.org/10.1093/nar/gkj067

4. Soetaert K, Petzoldt T. Inverse modelling, sensitivity and Monte Carlo analysis in R using package FME. J Stat Softw. 2010; 33: 1–28; http://doi.org/10.18637/jss.v033.i03

5. Soetaert K, Petzoldt T, Setzer RW. Solving differential equations in R: Package deSolve. J Stat Softw. 2010; 33: 1–25; http://doi.org/10.18637/jss.v033.i09

6. Soetaert K, Herman PMJ. A practical guide to ecological modelling. Using R as a simulation platform. 1st ed. Springer Dordrecht. 2009; http://doi.org/10.1007/978-1-4020-8624-3

7. Soetaert K. rootSolve: Nonlinear root finding, equilibrium and steady-state analysis of ordinary differential equations. 2009.

8. Plummer M, Best N, Cowles K, Vines K. CODA: Convergence diagnosis and output analysis for MCMC. R News. 2006; 6: 7–11.

9. Rohart F, Gautier B, Singh A, Le Cao K-A. mixOmics: An R package for ’omics feature selection and multiple data integration. PLoS Comput Biol. 2017; 13: e1005752; http://doi.org/10.1371/journal.pcbi.1005752

10. Nakazawa M. fmsb: Functions for Medical Statistics Book with some Demographic Data. R package version 0.7.5. 2023. Available from: https://CRAN.R-project.org/package=fmsb

11. Breuer O, Schultz A, Garratt LW, Turkovic L, Rosenow T, Murray CP, *et al*. *Aspergillus* infections and progression of structural lung disease in children with cystic fibrosis. Am J Respir Crit Care Med. 2020; 201: 688–696; https://doi.org/10.1164/rccm.201908-1585OC

12. Dios Caballero J de, Cantón R, Ponce-Alonso M, García-Clemente MM, Gómez G. de la Pedrosa E, López-Campos JL, *et al*. The human mycobiome in chronic respiratory diseases: Current situation and future perspectives. Microorganisms. 2022; 10: 810; https://doi.org/10.3390/microorganisms10040810

13. Tompkins MG, Pettit R. Beyond the guidelines: Treatment of allergic bronchopulmonary aspergillosis in cystic fibrosis. Ann Pharmacother. 2021; 56: 181–192; https://doi.org/10.1177/10600280211022065

14. Tracy MC, Okorie CU, Foley EA, Moss RB. Allergic bronchopulmonary aspergillosis. J Fungi. 2016; 2: 17; https://doi.org/10.3390/jof2020017

15. Benkerroum N. Aflatoxins: Producing-molds, structure, health issues and incidence in southeast Asian and sub-Saharan African countries. Int J Environ Res Public Health. 2020; 17: 1215; https://doi.org/10.3390/ijerph17041215

16. Kamala A, Shirima C, Jani B, Bakari M, Sillo H, Rusibamayila N, *et al*. Outbreak of an acute aflatoxicosis in Tanzania during 2016. World Mycotoxin J. 2018; 11: 311–320; https://doi.org/10.3920/WMJ2018.2344

17. Tan K. Aflatoxin and its toxic tragedies in Kenya. J Young Investig. 2020; 38; https://doi.org/10.22186/jyi.38.2.10-12

18. Chowdhary A, Agarwal K, Kathuria S, Gaur SN, Randhawa HS, Meis JF. Allergic bronchopulmonary mycosis due to fungi other than *Aspergillus*: A global overview. Crit Rev Microbiol. 2013; 40: 30–48; https://doi.org/10.3109/1040841X.2012.754401

19. Scurek M, Pokojova E, Doubkova M, Brat K. Allergic bronchopulmonary candidiasis: A review of the literature and a case report. BMC Pulm Med. 2022; 22: 1–6; https://doi.org/10.1186/s12890-022-01921-3

20. Green HD, Jones AM. Managing pulmonary infection in adults with cystic fibrosis: Adult cystic fibrosis series. Chest. 2022; https://doi.org/10.1016/j.chest.2022.02.007

21. Rayner R, Hiller E, Ispahani P, Baker M. *Haemophilus* infection in cystic fibrosis. Arch Dis Child. 1990; 65: 255–258; https://doi.org/10.1136/adc.65.3.255

22. Saliu F, Rizzo G, Bragonzi A, Cariani L, Cirillo DM, Colombo C, *et al*. Chronic infection by nontypeable *Haemophilus influenzae* fuels airway inflammation. ERJ Open Res. 2021; 7; https://doi.org/10.1183/23120541.00614-2020

23. Saliu F, Rizzo G, Bragonzi A, Cariani L, Cirillo DM, Colombo C, *et al*. The persistence of nontypeable *Haemophilus influenzae* fuels type 17 immunity in the lung. Eur Respir J 2021; 58: PA2107; https://doi.org/10.1183/13993003.congress-2021.PA2107

24. Rosales-Reyes R, Vargas-Roldán SY, Lezana-Fernández JL, Santos-Preciado JI. *Pseudomonas aeruginosa*: Genetic adaptation, a strategy for its persistence in cystic fibrosis. Arch Med Res. 2021; 52: 357–361; https://doi.org/10.1016/j.arcmed.2020.12.004

25. Watts SC, Judd LM, Carzino R, Ranganathan S, Holt KE. Genomic diversity and antimicrobial resistance of *Haemophilus* colonizing the airways of young children with cystic fibrosis. Msystems. 2021; 6: e00178–21; https://doi.org/10.1128/mSystems.00178-21

26. Carazo-Fernández L, González-Cortés C, López-Medrano R, Diez-Tascón C, Marcos-Benavides MF, Rivero-Lezcano OM. Mycobacterium avium complex infected cells promote growth of the pathogen *Pseudomonas aeruginosa*. Microb Pathog. 2022; 105549; https://doi.org/10.1016/j.micpath.2022.105549

27. Jamal F, Hammer MM. Nontuberculous mycobacterial infections. Radiol Clin North Am. 2022; 60: 399–408; https://doi.org/10.1016/j.rcl.2022.01.012

28. Lipner EM, Crooks JL, French J, Strong M, Nick JA, Prevots DR. Nontuberculous mycobacterial infection and environmental molybdenum in persons with cystic fibrosis: A case–control study in Colorado. J Expo Sci Environ Epidemiol. 2022; 32: 289–294; https://doi.org/10.1038/s41370-021-00360-2

29. Esther Jr CR, Esserman DA, Gilligan P, Kerr A, Noone PG. Chronic *Mycobacterium abscessus* infection and lung function decline in cystic fibrosis. J Cyst Fibros. 2010; 9: 117–123; https://doi.org/10.1016/j.jcf.2009.12.001

30. Sermet-Gaudelus I, Le Bourgeois M, Pierre-Audigier C, Offredo C, Guillemot D, Halley S, *et al*. *Mycobacterium abscessus* and children with cystic fibrosis. Emerg Infect Dis. 2003; 9: 1587; https://doi.org/10.3201/eid0912.020774

31. Mourad A, Baker AW, Stout JE. Reduction in expected survival associated with nontuberculous mycobacterial pulmonary disease. Clin Infect Dis. 2021; 72: e552–e557; https://doi.org/10.1093/cid/ciaa1267

32. Halstrom S, Price P, Thomson R. Environmental mycobacteria as a cause of human infection. Int J Mycobacteriol. 2015; 4: 81–91; https://doi.org/10.1016/j.ijmyco.2015.03.002

33. Victoria L, Gupta A, Gómez JL, Robledo J. *Mycobacterium abscessus* complex: A review of recent developments in an emerging pathogen. Front Cell Infect Microbiol. 2021; 11: 338; https://doi.org/10.3389/fcimb.2021.659997

34. Kim HJ, Kim IS, Lee S-G, Kim YJ, Silwal P, Kim JY, *et al*. MiR-144-3p is associated with pathological inflammation in patients infected with *Mycobacteroides abscessus*. Exp Mol Med. 2021; 53: 136–149; https://doi.org/10.1038/s12276-020-00552-0

35. Park EJ, Silwal P, Jo E-K. Host-pathogen interactions operative during *Mycobacteroides abscessus* infection. Immune Netw. 2021; 21; https://doi.org/10.4110/in.2021.21.e40

36. Bonyadi P, Saleh NT, Yamini M, Dehghani M, Amini K. Prevalence of antibiotic resistance of *Pseudomonas aeruginosa* in cystic fibrosis infection: A systematic review and meta-analysis. Microb Pathog. 2022; 105461; https://doi.org/10.1016/j.micpath.2022.105461

37. Biswas L, Götz F. Molecular mechanisms of *Staphylococcus* and *Pseudomonas* interactions in cystic fibrosis. Front Cell Infect Microbiol. 2022; 1383; https://doi.org/10.3389/fcimb.2021.824042

38. Fischer AJ, Singh SB, LaMarche MM, Maakestad LJ, Kienenberger ZE, Peña TA, *et al*. Sustained coinfections with *Staphylococcus aureus* and *Pseudomonas aeruginosa* in cystic fibrosis. Am J Respir Crit Care Med. 2021; 203: 328–338; https://doi.org/10.1164/rccm.202004-1322OC

39. Alcaraz E, Centrón D, Camicia G, Quiroga MP, Di Conza J, Rossi BP de. *Stenotrophomonas maltophilia* phenotypic and genotypic features through four-year

cystic fibrosis lung colonization. J Med Microbiol. 2021; 70: 001281; https://doi.org/10.1099/jmm.0.001281

40. Fluit AC, Bayjanov JR, Aguilar MD, Cantón R, Elborn S, Tunney MM, *et al*. Taxonomic position, antibiotic resistance and virulence factor production by *Stenotrophomonas* isolates from patients with cystic fibrosis and other chronic respiratory infections. BMC Microbiol. 2022; 22: 1–18; https://doi.org/10.1186/s12866-022-02466-5

41. Menetrey Q, Sorlin P, Jumas-Bilak E, Chiron R, Dupont C, Marchandin H. *Achromobacter xylosoxidans* and *Stenotrophomonas maltophilia*: Emerging pathogens well-armed for life in the cystic fibrosis patients' lung. Genes. 2021; 12: 610; https://doi.org/10.3390/genes12050610

42. Mojica MF, Humphries R, Lipuma JJ, Mathers AJ, Rao GG, Shelburne SA, *et al*. Clinical challenges treating *Stenotrophomonas maltophilia* infections: An update. JAC Antimicrob Resist. 2022; 4: dlac040; https://doi.org/10.1093/jacamr/dlac040
